# Cosurfactant-Induced Disorder in Polymersome Membrane Enhances Diffusion of Cargo Molecules

**DOI:** 10.1021/acsnano.6c00963

**Published:** 2026-04-29

**Authors:** Gabrielle A. Ong, Priyanka Sharan, Robert Graf, Kaloian Koynov, Yucong Chen, Arsh S. Hazrah, Katharina Landfester

**Affiliations:** Max Planck Institute for Polymer Research, Ackermannweg 10, 55128 Mainz, Germany

**Keywords:** polymersomes, cosurfactant, membrane packing, polymersome permeability, polymersome membrane analysis, GUV

## Abstract

Giant polymersomes are widely used as artificial cells due to their resemblance to natural cells; however, their intrinsically low membrane permeability remains a major limitation for applications such as drug delivery and bioreactors. Here, we report a microfluidic approach to fabricate permeable polymersomes using PB-*b*-PEO as a macromolecular surfactant and oleyl alcohol as a cosurfactant. Incorporation of the cosurfactant into the membrane induces membrane structural disorder in polymer packing, leading to enhanced membrane permeability. Using advanced nuclear magnetic resonance (NMR) spectroscopy, we directly probe the heterogeneity within polymersome membranes, representing membrane-level investigation in giant polymersomes. Comparison with polymersomes exhibiting tightly packed polymer assemblies reveals pronounced differences in the lateral mobility and permeability of the membrane. Our results demonstrate how subtle changes in formulation translate into distinct membrane structures, establishing a molecular basis for the rational design of polymersomes with tailored permeability.

## Graphical abstract

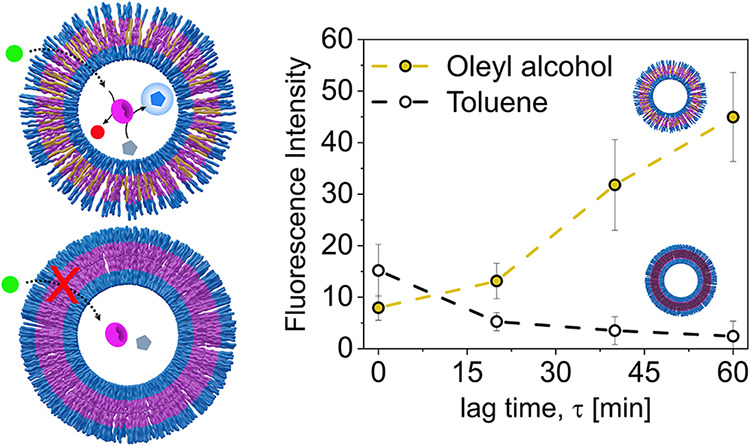

## Introduction

Polymersomes are increasingly recognized as cell mimics due to structural similarities between the cell membrane and the bilayer membrane formed from amphiphilic block copolymer in the former case.^1^ Three different populations of polymersomes exist: small unilamellar vesicles (SUVs) in the size range of 20–100 nm, large unilamellar vesicles (LUVs) in the size range of 100–1000 nm, and giant unilamellar vesicles (GUVs) with sizes >1 μm.^2^ In this study, we are focusing on GUVs; therefore, from this point on, the term polymersomes will refer to GUVs unless specified. The growing utilization of GUVs as artificial cells, or SUVs as nanoreactors and nanocarriers, owes to their chemical versatility, tunability, and higher mechanical stability.^3–5^ The hydrophilic core of polymersomes offers encapsulation of various aqueous components, thereby facilitating the hosting of various reactions ranging from biological transformations using enzymes to harsh organic processes involving photocatalysts.^6–8^ On the other hand, the bilayer membrane of the polymersomes acts as an isolating and protective layer, governing the exchange of molecules between the polymersome’s core and its surrounding.^9^ Therefore, the structural and mechanical properties of the bilayer membrane, which dictate membrane permeability, are central to their application. Additionally, the permeability of the membrane influences the operational capabilities of polymersomes as well as the stability of their encapsulated components.^10,11^ An overly restrictive membrane may limit substrate transmembrane diffusion, while excessive permeability undermines the stability of its enclosed species.^12^

Designing a permeable membrane and tuning the membrane properties of polymersomes require consideration of a variety of factors. For instance, membrane packing and lateral mobility are greatly influenced by the polymer’s molecular weight, structure, and membrane composition.^13^ Besides, the method and solvent used to assemble the polymersomes also dictate membrane properties. For instance, unlike the film rehydration method, where the formation of polymersomes is by rehydrating a thin film of polymer, in droplet microfluidics,^14^ and in the double emulsion dispersion (DED) method, the formation of polymersomes relies on solvent expulsion from double emulsions. Due to unfavorable interfacial tension, the organic solvent is removed from the double emulsions leaving the polymer behind, which self-assembles into a bilayer membrane.^15,16^ Highly volatile solvents, such as toluene or chloroform, are removed from the double emulsions via a combined process of partial dewetting and evaporation,^16^ and it is expected that a complete solvent removal can be achieved. On the other hand, depending upon solvent affinity and interfacial tensions, some alcohol-based solvents, which also act like cosurfactants, are more likely to be trapped in the hydrophobic region of the polymersome membrane,^17^ which can distort polymer assembly. Such distortion has been observed for hybrid membranes, where lipid and polymer are used to form bilayer membranes.^18,19^ The positioned cosurfactant might introduce structural disorder in the polymer packing and change membrane properties, in terms of membrane lateral mobility and permeability.

In order to comprehend the underlying differences in polymer chain organization in the membrane resulting from subtle changes during polymersome assembly methods, an extensive characterization of the membrane is required. Some hallmarks^20^ of characterization, such as size,^21^ morphology, membrane rigidity, surface charge, and stability, have been defined specifically for SUVs. However, the topology and dynamics of the polymer chains as a lamellar bilayer membrane using structure-resolving techniques have not been achieved. There are a few reports on the resolution of orientation topology of membrane-bound proteins in lipid-based bilayer membrane using magic-angle spinning (MAS) conditions for NMR spectroscopy experiments.^22^ However, elaborate membrane studies are largely missing in the case of LUVs, SUVs, and GUVs. In particular, the properties of GUVs with a thicker membrane and slower dynamic behavior fall outside the accessible range of many common characterization tools, therefore limiting our ability to directly measure key parameters that dictate transmembrane permeability.

In this study, we design two distinct polymersome membranes in terms of their membrane properties by selecting appropriate solvents in the polymersome assembly process. The choice of solvent has an impact on the solvent expulsion step, leading to the formation of polymersomes with different membrane properties and distinct permeability. When a cosurfactant is used as a solvent, part of it is retained in the membrane, inducing membrane structural disorder and hence enhancing membrane permeability. We utilize high-resolution NMR spectroscopy as the main tool to characterize the membrane dynamics of these distinct polymersomes. We characterize membrane heterogeneity and conformational mobility using high-resolution magnetic angle spinning (HR-MAS) to improve the spectral resolution. Moreover, we determine translational mobility of the polymer chains in the polymersome membrane via diffusion ordered spectroscopy (DOSY)^23^ under static conditions. The polymer membrane organization is further explored through sum frequency generation spectroscopy (SFG) experiments, which provide insights into the interactions between the polymer and water. Moreover, we corroborate the results from NMR spectroscopy using other well-known analytical tools for polymersomes. We utilized high-performance liquid chromatography (HPLC)^24,25^ to quantify polymersome membrane composition, and fluorescence correlation spectroscopy (FCS)^26^ and fluorescence recovery after photobleaching (FRAP)^27^ to obtain an estimate of relative polymer lateral mobility in the membrane. Finally, the understanding of the membrane properties is used to characterize differences in the transmembrane diffusion of a variety of cargoes across the membrane in the solvent-tuned polymersomes. The study gains structural insights into the polymersome membrane, which is required to tailor optimal polymersome design for targeted applications.

## Results and Discussion

Polymeric giant unilamellar vesicles (polymersomes) are assembled using droplet microfluidics. Briefly, a 3-inlet microfluidic system is used to fabricate water–oil–water double emulsions. Typically, the inner phase, the phase that must be encapsulated inside the polymersomes, and the outer phase, the phase in which these polymersomes are dispersed, consisted of aqueous solutions. The middle phase consisted of poly­(butadiene)_22_–poly­(ethylene oxide)_14_ (PB-*b*-PEO, with *M*_n_ (PB) = 1200 g/mol and *M*_n_ (PEO) = 600 g/mol), a membrane-forming macromolecular surfactant^1^ dissolved in an organic solvent at a concentration of 10 mg/mL. Depending upon the solvent used, the solvent removal occurs distinctly, which might influence the membrane properties of the polymersomes. Either oleyl alcohol or toluene is used as the organic solvent to dissolve the polymer, where the polymer chains self-assemble via hydrophilic–hydrophobic phase separation. Oleyl alcohol not only acts as a solvent but also functions as a cosurfactant due to the amphiphilicity introduced by the hydroxyl group. When oleyl alcohol is used as the solvent, the solvent dewets from the double emulsion, leading to a prompt transition to polymersomes. Roughly within a minute of production, polymersomes start to appear. However, due to a similar long hydrocarbon chain structure with unsaturation in PB and oleyl alcohol, there could be an affinity of some amount of oleyl alcohol to stay in the hydrophobic part of the membrane. This retention of oleyl alcohol is confirmed using HPLC, where roughly 1–6% w/w of oleyl alcohol can be found in the polymer membrane (Table S1).^10^ The presence of oleyl alcohol may generate structural disorder in the polymer membrane packing, resulting in a “leakier” polymersome membrane as shown in Figure 1a. On the other hand, in the case of double emulsions produced using toluene as the solvent, a more complex solvent removal procedure occurs whereby a combination of dewetting and solvent evaporation leads to the formation of polymersomes.^16^ In the first step, a partial dewetting of the toluene phase results in the formation of a lens of the toluene phase, making a nonzero contact angle with the membrane. In the next step, a complete toluene evaporation results in the formation of polymersome and the excess polymer is left as a patch on the polymersome surface (Figures S2 and S3). This process is hypothesized to form better polymer packing shown in Figure 1b, forming a more ordered polymer membrane and hence limited membrane permeability. In the confocal microscope (Figure S4a), the two different types of polymersomes: oleyl alcohol-based polymersomes (OAP) and toluene-based polymersomes (TP) appear similar, except for the polymer patch in the case of TP as excess material on one side of the polymersome (Figure S4b, bottom left side). To validate our hypothesis of a “disordered” membrane in the oleyl alcohol and an “ordered” membrane in the toluene case, we start by characterizing the membrane using NMR spectroscopy methods.

**Figure 1:**
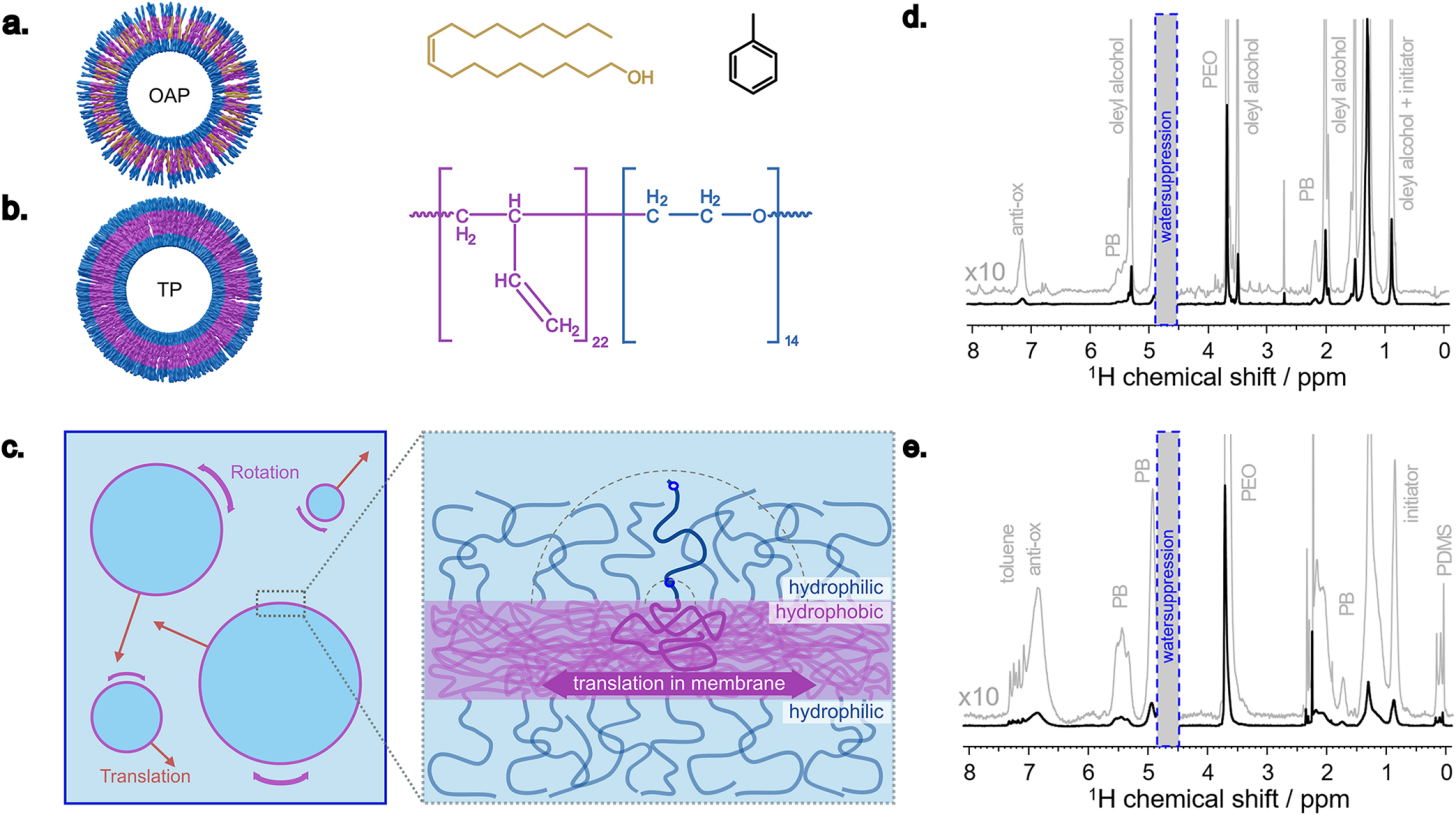
(a) OAP and (b) TP structural illustration where the blue-purple chains represent polymer and the yellow chains represent oleyl alcohol. (c) Schematic illustration of polymer movement as polymersomes in bulk and (zoomed-in) in the interface of polymersome membrane. HR-MAS NMR spectra of (d) OAP and (e) TP showing membrane compositions.

NMR characterization for such thin membranes remains technically demanding: the isotropic Brownian motion of dissolved polymer chains is strongly reduced by the formation of the polymersome membrane, and residual molecular fluctuations superimpose with the rotational and translational motion of the vesicles reducing the spectral resolution of NMR spectroscopy in solution, which capture overall molecular rotation motion as changes in line width (Figure 1c). Additionally, the size distribution of the vesicles obtained during polymersomes handling leads to a very heterogeneous dynamic behavior in the NMR measurements. The polymer segments of smaller-sized vesicles (obtained due to unintended polymersome rupture) are observed with reasonable spectral resolution, as compared to micrometer-sized polymersomes. For polymersomes in the 50 μm size range, the overall rotation of the vesicle is seen on the molecular level as a translational motion, while local molecular fluctuations determine the *T*_2_ relaxation time, leading to a heterogeneous distribution of *T*_2_ relaxation times. To overcome these challenges, NMR experiments have been performed under high-resolution magic-angle spinning (HR-MAS) conditions. On the other hand, diffusion ordered spectroscopy (DOSY) NMR measurements have been performed under static condition, in order to avoid the influence of centrifugal forces on the polymers lateral mobility along the membrane processes. Figure 1d,e show the HR-MAS NMR spectra of the OAP and TP, respectively. The NMR spectrum of the OAP shows a significant amount of oleyl alcohol in the membrane, which can be observed via the peak at 3.5 ppm in Figure 1d, assigned to the proton signal of the CH_2_ site right before OH at the chain end of oleyl alcohol. Another peak belonging to the CH_3_ group of the oleyl alcohol can be observed at around 0.9 ppm, though overlapping with a peak assigned to the initiator (butyllithium) used in polymer synthesis. On the other hand, in the case of HR-MAS NMR spectra for TP, only very weak toluene signals are detected around 7–7.3 ppm (Figure 1e). From the NMR spectra shown, it is not possible to localize the remaining toluene. Probing spatial proximity on the molecular level using NOESY NMR experiments, even at the longest mixing times, did not show any correlation peaks between the toluene signals and other ^1^H sites of the polymer (Figure S5). This suggests that the observed traces of toluene reside in the deuterated aqueous phase. In freshly prepared double emulsions where the toluene is still present in the membrane, two toluene peaks are observed (Figure S6). These experiments indicate that in double emulsions, toluene molecules reside in two distinct chemical environments: toluene in the membrane and toluene in the aqueous phase. During the conversion of double emulsions to polymersomes, toluene is completely removed from the membrane, resulting in a single distinct peak arising from residual toluene in the aqueous phase.

Next, we looked into the lateral diffusion behavior of the polymers. From DOSY NMR measurements, the translational diffusion coefficients of the membrane components and solvent molecules are determined. Figure 2a,2b shows the diffusion coefficients of the PEO and PB parts in both OAP and TP to be around 10^–11^ m^2^ s^–1^. The matching segmental diffusion coefficient of the polymer components and the oleyl alcohol supports the assumption that the leftover oleyl alcohol is bound to the polymersome membrane (Figure 2a). In a phase-separated system, the superposition of local molecular diffusion and overall diffusion would differ. While in a non-phase-separated system, the overall diffusion of the components has to match by definition and local molecular diffusion coefficients will come closer. In our measurements, the recorded values for the polymer segments are very similar to those of the oleyl alcohol. This happens because the most mobile molecular component acts as a lubricant for the other components and is, at the same time, hampered in its own molecular mobility. Another affecting parameter is the size distribution of the polymersomes, which complicates the interpretation of the measured diffusion coefficients. In the case of TP, the diffusion coefficient of toluene is 2 orders of magnitude higher than that of the polymer part (Figure 2b). These considerably different diffusion coefficient values indicate a lack of interaction between toluene and the polymer, implying that toluene can be fully removed from the double emulsions to yield a solvent-free polymer structure in the TP case.

**Figure 2:**
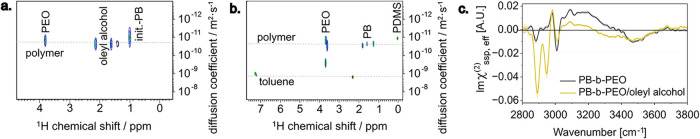
Polymer–solvent interaction in the polymersome membrane. DOSY spectra of (a) OAP and (b) TP displaying the diffusion coefficient of each membrane component. (c) The measured imaginary part of the HD-SFG spectra (Im χ^(2)^_ssp,eff_) of the PB-*b*-PEO and PB-*b*-PEO/oleyl alcohol interface.

Another important observation of Figure 2a,2b is the broad distribution of diffusion coefficient values of the PEO and PB part in the membrane, compared to DOSY measurements of the polymer dissolved in THF (Figure S7b). This indicates that the local segmental mobility varies along the polymer chain in a membrane, and the size distribution of vesicles can also have an effect (Figure 1c). Looking back at Figure 1c, the polymersome membrane contains a hydrophilic–hydrophobic interface region. The polymer segment positioned near the interface is more restricted in its rotational mobility compared to the polymer segment at the chain end. Additionally, traces of solvent located at the interface can create local swelling, increasing the molecular mobility of components in a certain region. Therefore, the integration values of the polymer peaks do not quantitatively reflect the building block composition of our polymersomes. On the other hand, we are now able to understand much better the complexity and heterogeneity of polymersome membranes at a molecular level.

To further probe the interfacial structure, we employed heterodyne-detected vibrational sum frequency generation (HD-SFG) spectroscopy. HD-SFG spectroscopy is a phase-resolved nonlinear optical technique that reveals the complex second-order susceptibility, providing direct access to both the magnitude and sign of the interfacial response.^28^ Its inherent surface specificity makes it ideally suited for hydrated polymer membranes, where it directly probes the orientation and hydrogen bonding network of interfacial water, similar to its established use at lipid and surfactant monolayers at the water–air interface.^29–31^ Here, the OH stretch region reports on the interfacial interaction of the water molecules with the polymers, while the CH-stretch region reflects hydrophobic chain packing and order. For the HD-SFG measurements, a polymer–CHCl_3_ mixture was drop cast onto the water surface to form a monolayer at the air–water interface, with solvent evaporation leaving a thin interfacial polymer film. To model the OA membrane, oleyl alcohol was added to the polymer-CHCl_3_ mixture and drop cast as described above.

The SFG spectra (Figure 2c) reveal distinct interfacial structures between the two systems. The pure polymer membrane shows a stronger and more structured OH band with a pronounced positive band around 3100–3300 cm^–1^, consistent with more strongly oriented interfacial water. In contrast, the polymer layer with oleyl alcohol shows a diminished positive OH band in the 3100–3300 cm^–1^ region that shifts to a broader negative feature between 3250 and 3500 cm^–1^, indicating a redistribution toward weaker, less ordered hydrogen bonding and a change in the net orientation of interfacial water molecules. These observations suggest altered intermolecular interactions between interfacial water and the polymer, reflecting a less ordered hydration structure at the membrane surface. These HD-SFG results corroborate the NMR spectroscopy and diffusion data, confirming that molecular-level disorder in the oleyl alcohol-containing polymer layer disrupts interfacial ordering, leading to enhanced permeability. In the CH-stretch region below ∼3000 cm^–1^, the polymer membrane without oleyl alcohol exhibits a weak response, consistent with a low net orientation of hydrocarbon groups at the interface. Upon incorporation of oleyl alcohol, the CH-stretch intensity increases. This signal can be attributed primarily to the alkyl chains of oleyl alcohol itself, which introduce additional CH oscillators with a measurable orientational contribution at the interface. Importantly, this increase reflects the presence and orientational contribution of oleyl alcohol at the interface and does not by itself imply changes in packing or polymer organization.

To corroborate the NMR data and further validate our hypothesis, we assessed the dye molecules’ lateral mobility within the membrane by using fluorescence correlation spectroscopy (FCS). FCS measures fluorescence intensity fluctuations arising from the translational diffusion of fluorescent molecules through a small observation volume (typically the focus of a confocal microscope) and is widely applied to quantify diffusion processes in lipid membranes.^32^ For this purpose, a hydrophobic dye (Nile red) dissolved in DMSO was added to polymersome suspensions. Owing to its hydrophobicity, the dye partitions into the nonpolar regions of the polymersome membrane (Figure 3a). The diffusion time (τ*_D_*) required for the dye to traverse the observation volume was extracted from the FCS curves and serves as a proxy for membrane dynamics, with shorter τ_D_ indicating higher lateral mobility. As shown in Figure 3b and Table S2 in the SI, Nile red diffuses almost three times faster in the OAP membranes (τ*_D_* ≈ 1500 μs) than in the TP membranes (τ*_D_* ≈ 4200 μs). These results indicate that OAP membranes are considerably more fluid-like, influenced by the structural disorder generated by residual oleyl alcohol swelling the PB-*b*-PEO matrix, whereas TP membranes, devoid of residual solvent, exhibit a more rigid structure.

**Figure 3:**
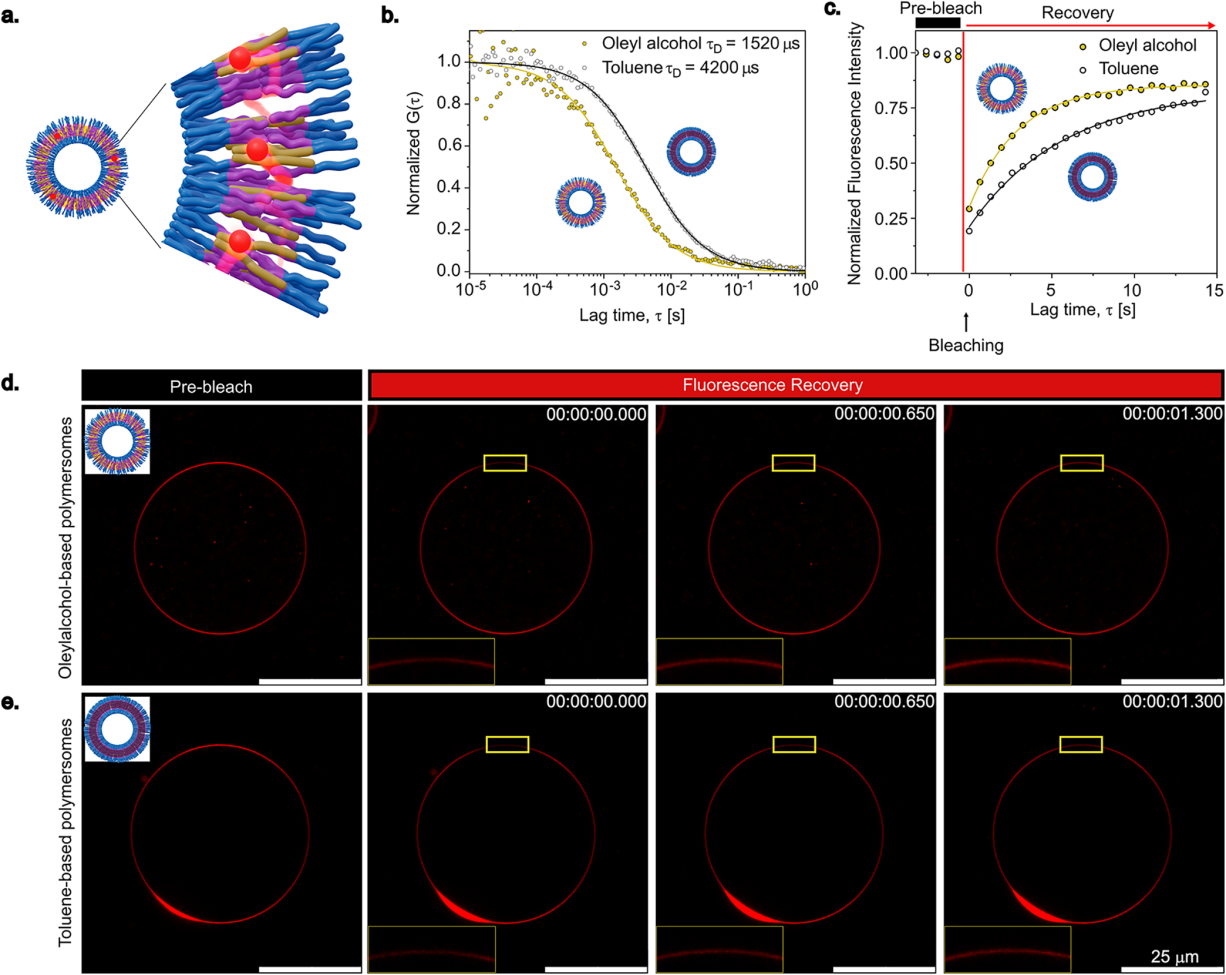
Assessing membrane lateral mobility in the polymersomes. (a) Schematic representation of Nile red diffusion in polymersome membrane used for FCS and FRAP measurement. Red spheres represent Nile red. (b) Normalized experimental FCS autocorrelation curves (symbols) and the corresponding fits with eq 1 (lines), yielding the diffusion time of Nile red. (c) Fluorescence intensity measurement during the FRAP experiment, displaying the slower fluorescence recovery of Nile red in the TP membrane compared to the OAP membrane. Time-lapse confocal images captured during the FRAP experiment in case of (d) OAP and (e) TP, confirming the slower fluorescence recovery of the bleached area (inset) in the case of TP. Results are representative of one polymersome.

In order to further substantiate our observations, fluorescence recovery after photobleaching (FRAP) experiment is conducted in the membrane of the two polymersomes. In this method, a localized region within the polymersome membrane containing a small amount of Nile red dye is photobleached using a high-intensity laser beam, and the subsequent fluorescence recovery is recorded. Depending on the lateral mobility of the membrane, the kinetics of fluorescence recovery would be different as the bleached dye molecules diffuse out and the nonbleached dye molecules diffuse into the exposed area. As can be seen in Figures 3c and S4c, the fluorescence intensity recovers faster in the case of OAP than in TP. The faster fluorescence recovery of the OAP as compared to the TP is also evident in the time-lapse confocal images in Figure 3d,3e. These results correlate well with FCS data, suggesting that the dye molecule can diffuse faster in the case of the OAP, further implying a more fluid-like membrane in this case.

A less fluid-like membrane in the case of TP would mean a less leaky polymersome as compared to OAP, which has been demonstrated to be intrinsically permeable to a variety of small molecules.^10^ Based on the target application, these distinct characteristics of the membrane permeability originating from the structural disorder within the membrane due to the presence of a dopant, in this case a cosurfactant (oleyl alcohol), must be accounted for, and accordingly, an appropriate solvent must be selected. Therefore, in the next step, a variety of small molecules are tested for their transmembrane diffusion across the polymersome membrane.

To better isolate the differences in the membrane properties of the two different types of polymersomes, the transmembrane diffusion of small ions through the membrane is studied. At first, the diffusivity of protons across the membrane is measured via changes in the fluorescence intensity of a pH-sensitive molecule, 8-hydroxypyrene-1,3,6-trisulfonic acid (HPTS). The pH probe was encapsulated inside the polymersomes (Figure 4a). The pH of the environment dictates the fluorescence intensity of the HPTS molecule. In an acidic environment, the fluorescence is low, whereas in a basic pH, it shows strong fluorescence. When the HPTS encapsulated polymersomes are immersed in baths of outer fluid solutions at different pH values, it is evident that the fluorescence intensity scales with the pH of the surrounding in both OAP and TP, indicating transmembrane diffusion of protons in both (Figure 4b,4c). However, when looking at the kinetics of proton diffusion across the membrane and the subsequent change in the fluorescence intensity, the difference between the two types of membranes in proton permeability becomes visible. In the case of the OAP, a fast change in fluorescence intensity is witnessed as the polymersomes are immersed in the outer fluid pH baths. On the other hand, TP displayed a slower intensity variation (Figure S8). To highlight the delay in proton transmembrane diffusion in the case of TP, the temporal evolution of the fluorescence intensity inside the polymersomes is quantified using ImageJ (Figure 4d). Even after 30 min, the fluorescence intensity continues to develop at both acidic and basic pH, revealing much slower proton transmembrane diffusion kinetics in the case of TP.

**Figure 4:**
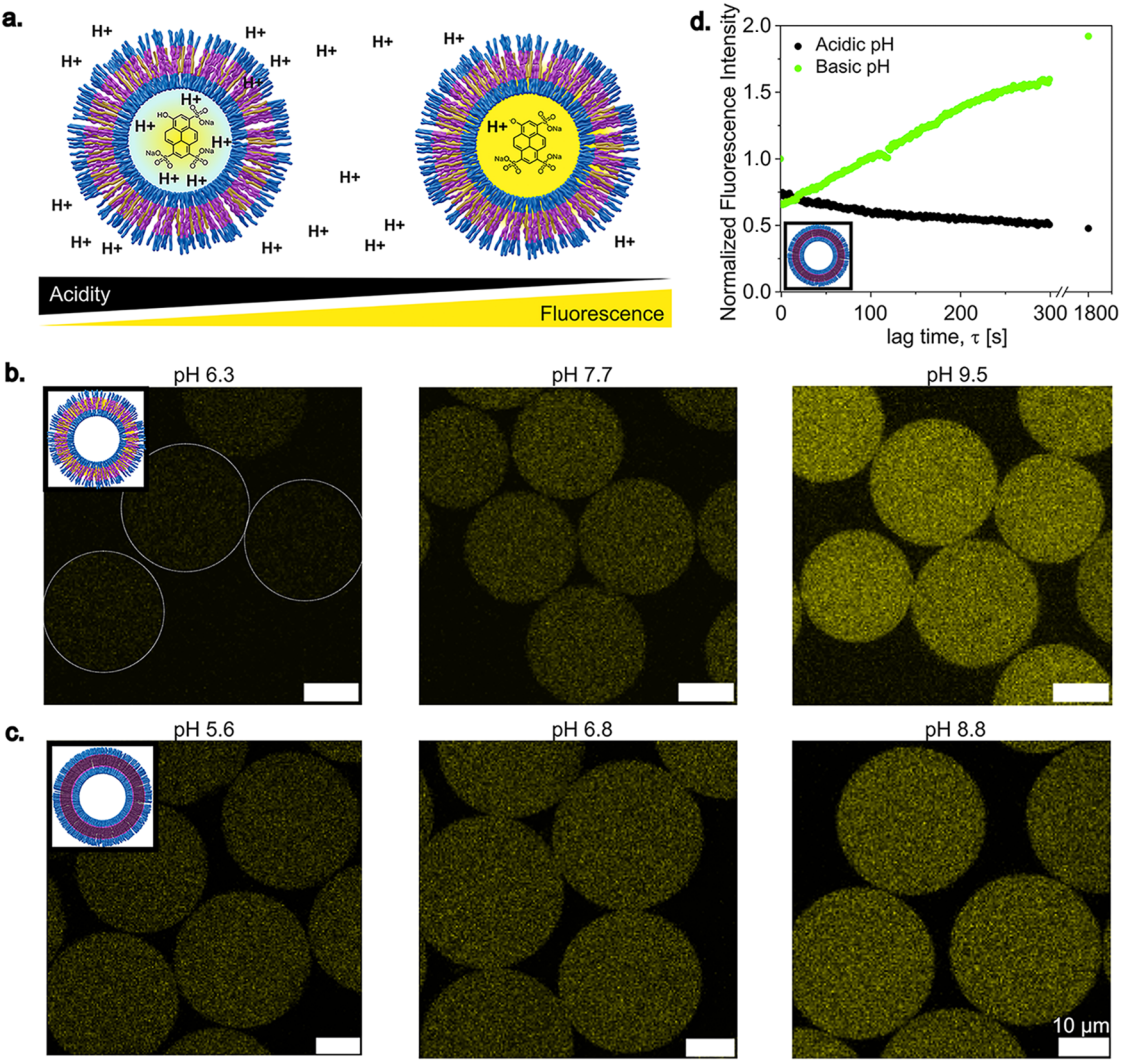
Proton permeability across polymersome membrane. (a) Schematic representation of HPTS molecule encapsulated inside polymersomes, and the change in the fluorescence intensity depending on the pH of the environment. (b) Fluorescence image of HPTS-encapsulated OAP in different pH bath. (c) Fluorescence image of HPTS encapsulated TP in different pH bath. (d) Gradual fluorescence intensity changes of HPTS-encapsulated TP when incubated inside acidic and basic baths. Fluorescence intensity was measured using ImageJ by averaging five polymersomes within one batch.

To emphasize further the role of cosurfactant-mediated membrane tuning, the diffusivities of several biologically relevant ions across the membrane of the OAP and TP are studied. While it is challenging to directly visualize the transmembrane diffusion of ions by means of fluorescence, here an indirect method is selected. Briefly, complex coacervates formed using polyelectrolyte pairs, in this case carboxy-amylose (amylose-COOH) and diethylaminoethyl-dextran (DEAE-dextran) and a rhodamine B isothiocyanate (RITC)-labelled DEAE-dextran, are encapsulated inside polymersomes during microfluidic production. As complex coacervation is an electrostatically driven liquid–liquid phase separation between oppositely charged polyelectrolytes, the overall formation is strongly dependent upon the pH and salt concentration. Due to the interaction of oppositely charged polyelectrolytes, a loss of configurational entropy results, which is counterbalanced by the release of counterions. Therefore, the system’s final entropy is highly dependent on the release of counterions, which is one of the key reasons why complex coacervation is sensitive to the ionic strength of the medium.^33^ In the bulk, the dependence of a variety of salt solutions on the coacervate formation is tested. Briefly, the already formed coacervates are cast as a droplet on a glass slide, and the dissolution of the coacervate droplets is monitored using confocal microscopy after salt addition. In both monovalent and divalent salts, the coacervate droplets appear to dissolve rapidly once the critical ionic strength is achieved (Figures S9 and S12).

To study the permeability of ions across the polymersome membrane, polymersomes with coacervates encapsulated are immersed in baths of different salt solutions (Figure 5a). When no salt is added, the coacervates appear as distinct and stable liquid droplets inside the polymersomes (Figure 5b,5c, no salt). When they are immersed in baths of salt solution, their stability is affected depending on whether the ions can diffuse across the polymersome membrane. In the case of OAP, in all the salt solutions studied, the coacervate droplets seem to dissolve, pointing at transmembrane transport of ions in this case (Figures 5b,5d and S10a–e). A closer look at the kinetics of coacervate dissolution in different salt solutions in the OAP reveals that in the case of divalent salts such as MgCl_2_ and CaCl_2_, the dissolution is much faster on the scale of 20–40 min. Whereas, in the case of monovalent salts such as LiCl, NaCl, and KCl, the dynamics is quite slow in the range of 3 to 6 h until the coacervate dissolves completely (Figure S10a–e). The observed trends of a higher effect of divalent salts than the monovalent salts on coacervate dissolution correlate well with the Hofmeister series.^33^ On the other hand, in the case of TP, the coacervate droplets are stable within 24 h of imaging (Figure 5c,d, and 52 h of imaging in Figure S11a–e), suggesting little to no ion permeability in this case. However, it should be mentioned that these experiments are indirect measurements and give a qualitative picture of ion transmembrane transport. It is challenging to quantify the permeability of small/negligible number of ions that might be diffusing across the membrane because this is below the critical ionic strength required for coacervate dissolution (Figure S12).

**Figure 5:**
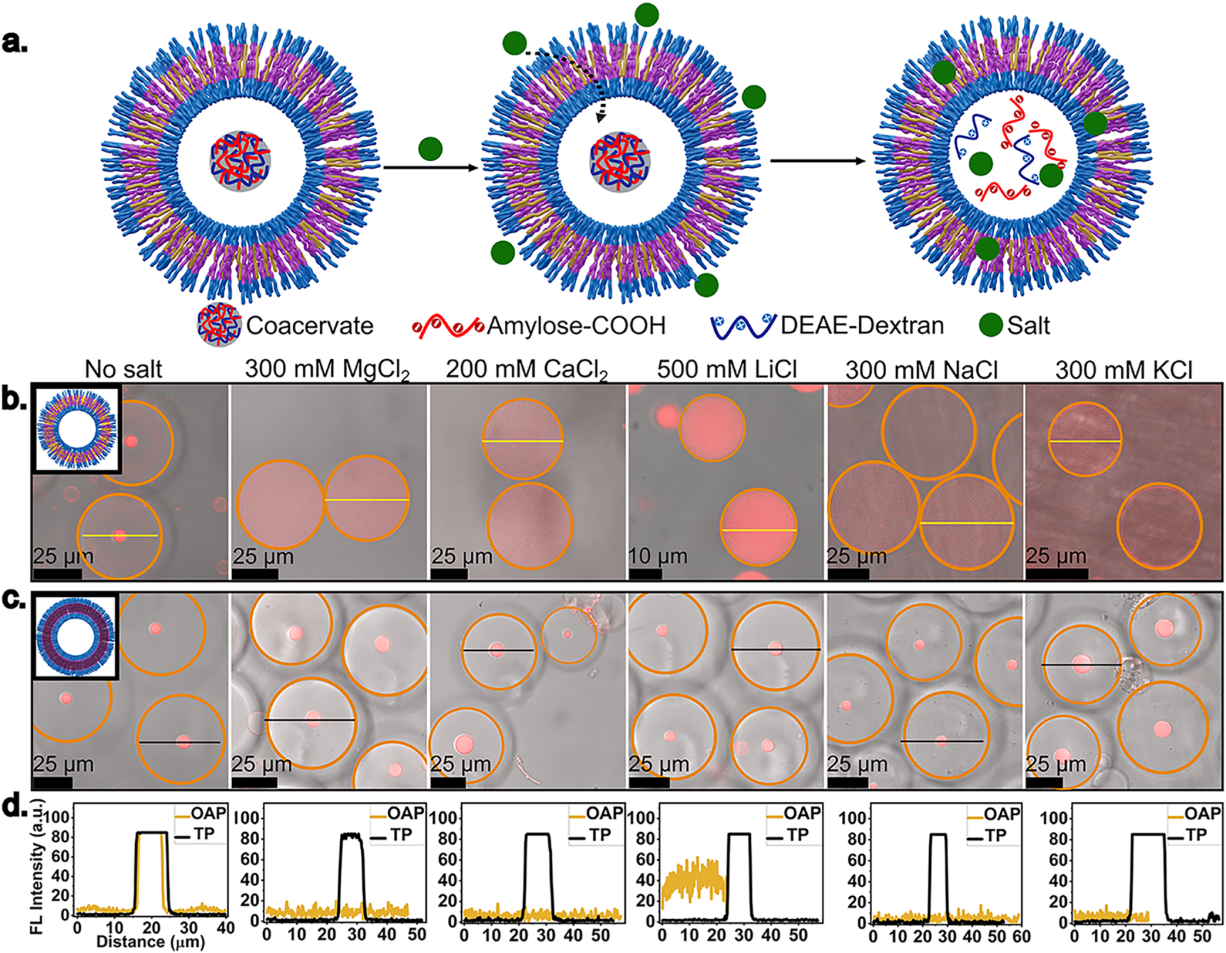
Ion permeability across polymersome membrane: (a) Schematic illustration of the coacervate encapsulated polymersomes, followed by ion transmembrane diffusion and subsequent coacervate dissolution. (b) Coacervate encapsulated OAP polymersomes after different time intervals in various salt concentrations. (c) Coacervate encapsulated TP after 24 h in different salt concentrations. (d) Fluorescence intensity profile showing the distribution of RITC-labelled DEAE-dextran-inside OAP and TP in (b) and (c). The fluorescence profiles are plotted along the yellow (in b) and black (in c) lines.

In order to emphasize the role of solvent and the subsequent transmembrane diffusion of cargoes, a model polymersome microreactor system is designed by encapsulating glucose dehydrogenase (GDH) enzyme and NAD^+^ cofactor (Figure 6a). When the substrate of the enzyme, in this case glucose, is supplied to the enzyme via transmembrane diffusion, the substrate is converted into gluconic acid, and the cofactor is transformed into NADH, a fluorescent molecule typically excited in the UV region. Fluorescence of NADH in the UV region can be detected using a DAPI filter in a fluorescence microscope.^34^ In the case of OAP, a steady increase in the fluorescence intensity inside the polymersomes confirms transmembrane permeability of glucose (Figure 6b,6d). On the other hand, in the case of TP, no increase in the fluorescence can be detected, suggesting no glucose permeability through toluene-based polymersome membranes (Figure 6c,6d). The remaining oleyl alcohol left in the OAP renders them inherently permeable and makes them particularly suitable as microreactor systems where non-gated transport is required. On the other hand, these experiments particularly highlight the influence of the toluene solvent in the membrane formation and the subsequent impermeability of the polymersome. The TP is especially suitable for systems where fine control or gated transport is necessary. Certainly, it is challenging to achieve gated transport using pristine toluene polymersomes; however, it can be realized by incorporating stimuli-responsive materials in the membrane.^6^

**Figure 6:**
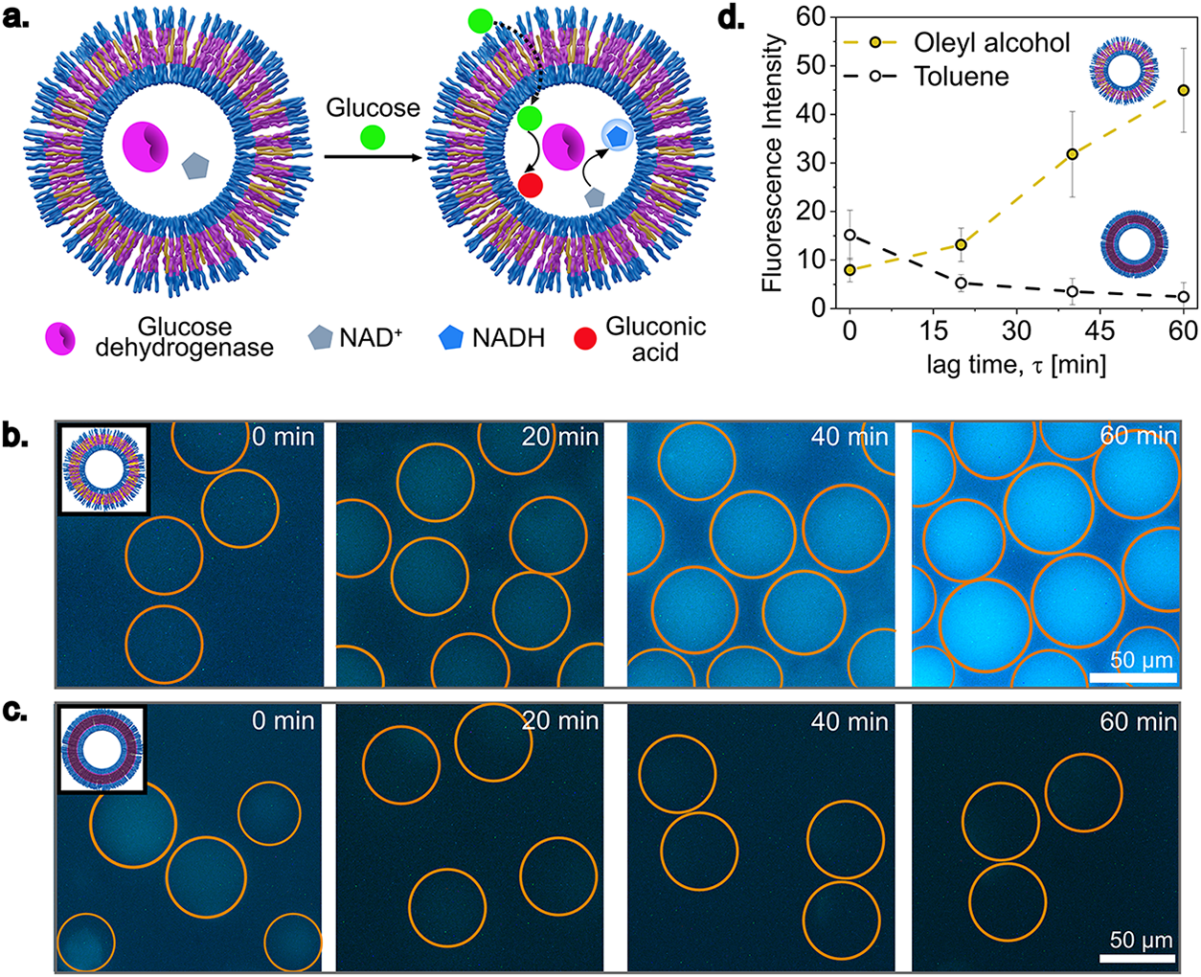
Glucose permeability across polymersome membrane: (a) Schematic illustration of GDH reaction in polymersomes with the substrate (glucose) fed from outside. The internal reaction produces NADH, which has fluorescence. (b) Time-lapse fluorescence images of the GDH reaction inside OAP. (c) Time-lapse fluorescence images of the GDH reaction inside TP. (d) Fluorescence intensity change over time measured using ImageJ. Fluorescence intensity averaged from five polymersomes within the same batch.

## Conclusions

We achieve permeable polymersome membranes by selecting the appropriate solvent in the polymersome assembly step, effectively changing the structure of the polymer membrane. The expulsion of the solvent from the double emulsion to form polymersomes has important implications for the final bilayer assembly. Using a more volatile solvent such as toluene, a complete solvent removal can be achieved via a combination of partial dewetting and solvent evaporation. On the other hand, when oleyl alcohol with dual function as solvent and a cosurfactant is used, part of it is retained in the membrane’s hydrophobic region. We use NMR spectroscopy, FCS, FRAP, SFG, and HPLC to comprehend the differences in the bilayer assembly when these two different solvent expulsion mechanisms occur. We showed that when toluene is used as the solvent, the membrane is mainly composed of the polymer; whereas, when using a cosurfactant (oleyl alcohol) as the solvent, the membrane is built from a combination of the polymer and some amount of retained cosurfactant. The retained cosurfactant in the membrane induces structural disorder, which affects the overall property of the polymersome membranes and hence, the membrane permeability. The retained cosurfactant influences permeability in the polymersome membranes, facilitating a non-gated transport in this case. Whereas, in the toluene polymersome, the membrane is tightly packed and results in limited transmembrane permeability of molecules, which is true even for small ions. We choose biologically relevant ions, such as Na^+^, Mg^2+^, and Ca^2+^, to highlight the relevance of selecting an appropriate solvent when assembling the polymersome. The assay used to detect transmembrane diffusion of ions indicates little to no ion diffusion in the case of TP. However, the assay used here is an indirect readout, and the ion transmembrane diffusion could be below the detection limit of the assay. Due to the impermeable nature of toluene polymersomes, they become highly relevant when a gated transport is necessary for specific applications.^11^ This study highlights the importance of studying the polymersome membrane using structure determination tools to comprehend the differences arising from subtle changes in the ingredients forming the membrane. The knowledge of the membrane properties is specifically beneficial for tailoring application-oriented polymersome design. In general, the design of such cosurfactant-retaining permeable polymersome systems depends on the interaction between the polymer and the cosurfactant. In the PB-*b*-PEO/oleyl alcohol platform, due to a similar long hydrocarbon chain structure and unsaturation, there is an affinity of the oleyl alcohol to get retained in the membrane. Therefore, in order to design a similar system using other polymers and cosurfactants, careful consideration of these factors must be made. In this work, we focused on the microfluidics technique. However, with other polymersome fabrication techniques, such as the double emulsion droplet (DED) method, where polymersome formation relies on solvent expulsion from double emulsions, the same surfactant-retaining polymersome system could be established.

## Experimental Section

### Microfluidic Setup

Microfluidic channels were produced as follows: SU-8 photoresist positive relief channels on silicon wafers were used as the masters. Next, PDMS and curing agent in a 9:1 ratio were mixed well and then degassed using centrifugation. This mixture was poured on top of the silicon wafer stored in a glass Petri dish. Any bubbles appearing in the PDMS during the pouring process were popped by using a sharp needle. In the next step, the PDMS was cured at 80 °C for at least 2 h, after which PDMS pieces containing individual channels were cut out using a scalpel. Using a biopsy needle, holes were punctured in the PDMS at the end of each channel to mark the inlet and outlet connections. Next, the coverslip was cleaned using isopropanol and pressurized air. The glass slide and the PDMS pieces were activated using a plasma cleaner for 30 s at 20% radiofrequency (RF) and were bonded together by gently pressing the PDMS piece on the glass slide. Immediately after the bonding, the outer channel was coated with 3% w/v poly­(vinyl alcohol) (PVA) using vacuum. The PVA solution was connected to the outer inlet channel, and vacuum was connected to the outlet channel. The PVA was flowed through the outer channel for 4 min, and finally the chip was baked at 80 °C for at least 2 h.

### Glass Coating of the Microfluidic Chips

Glass precursor solution was prepared following the procedure from Abate et al.^35^ Briefly, tetraethoxysilane, methyltriethoxysilane, ethanol, and pH 4.5 adjusted water were combined in a 1:1:1:1 ratio and heated at 65 °C for 3 h while stirring. Next, unbonded PDMS pieces with punched holes were plasma cleaned for 30 s at 20% RF with the channels facing up to activate the surface. Immediately, on top of the channel, the above-prepared glass precursor solution was poured. It was also ensured that this solution filled the holes to coat them properly with glass. In the next step, the excess glass precursor solution was removed by using pressurized air instantly. Next, the chips were placed on a hot plate at 105 °C for 30 min to initiate the gelation process. Finally, these PDMS pieces were used for making microfluidic chips, whereby the usual procedure of bonding to a glass slide and subsequent coating with PVA was carried out.

### Microfluidic-Assisted Formation of Polymersomes

Polymer solutions were prepared by dissolving 10 mg of poly­(butadiene)_22_–poly­(ethylene oxide)_14_ (*M*_n_ = 1200-*b*-600 g mol^–1^) (PB-*b*-PEO) polymer in 1 mL of organic solvent, either oleyl alcohol or toluene. The inner and outer fluids were 100 mM Na_2_SO_4_ and 300 mM NaCl, respectively. The inner fluid, polymer solution, and outer fluid were drawn into 1 mL plastic syringes connected to a needle. Air bubbles were manually expelled from the syringes. The syringes were fixed on a Cetoni Nemesys microfluidic pump system, and the microfluidic chip was placed under a microscope. PTFE tubings were used to connect the syringes with the appropriate inlets as well as to connect the outlet with a collecting vial. Production was initiated by applying flow rates of 100 μL h^–1^ for all solutions. Once all of the solutions reached the channels, the flow rates were adjusted as needed. The vesicles were discarded until the double emulsion at the second junction has stabilized. The double emulsions were collected in an Eppendorf tube for dewetting and further analysis.

### Nuclear Magnetic Resonance Spectroscopy

The polymersomes were prepared in the same way as previously described. In both inner and outer fluid, H_2_O was exchanged with D_2_O. For OAP, the samples were probed within the same day as polymersomes production. For TP, the samples were first incubated for 1 week, before they were used for NMR sample preparation. Around 60 μL of polymersomes were collected from the bottom of each collection tube to obtain concentrated polymersomes. The “pure” polymersomes were then transferred to 4 mm MAS NMR rotors for HR-MAS NMR measurement. For the diffusion analysis, 20 μL of the concentrated polymersomes was added into an NMR tube filled with 500 μL of 300 mM NaCl in D_2_O. The sample was analyzed using DOSY experiment, in order to obtain diffusion information on components in the polymersomes.

The DOSY NMR measurements have been performed at 700.25 MHz ^1^H Larmor frequency using a Bruker Avance III console and a commercial three-channel inverse (TXI) high-resolution probe with a pulsed gradient field of <0.55 T/m. Although the polymersome samples were prepared with pure D_2_O (99.8% D), the water signal had to be suppressed using optimized presaturation schemes in order to observe polymer signals with their relatively short *T*_2_ times. HR-MAS NMR measurements have been performed at 850.23 MHz ^1^H Larmor frequency using a ^1^H/^13^C/^15^N HR-MAS Bruker iProbe connected to an Avance NEO console at a 5 kHz MAS spinning frequency. Despite the higher polymersome concentration in the HR-MAS measurements, solvent signal suppression via presaturation of the water signal was required to record the shown ^1^H NMR spectra.

### Vibrational Sum Frequency Generation Spectroscopy

The detailed specifications of the HD-SFG spectrometer have been described elsewhere.^36,37^ Briefly, SFG measurements were performed in a collinear beam geometry using a Ti:sapphire laser system (Spectra-Physics; pulse duration ∼40 fs, pulse energy 5 mJ, repetition rate 1 kHz). A portion of the output was directed to a pulse shaper to generate a narrowband visible pulse centered at ∼800 nm (bandwidth ∼15 cm^–1^), while the remaining output pumped a commercial optical parametric amplifier (TOPAS SHBS-400, Spectra-Physics) to produce a broadband mid-infrared pulse centered at ∼3000 cm^–1^. The visible and mid-IR pulses were sequentially focused onto a y-cut quartz crystal to generate a local oscillator and then onto the sample surface at an incident angle of 45° after passing through a 2 mm SrTiO_3_ phase plate. The emitted SFG signal interfered with the local oscillator to produce an interferogram, which was recorded using a liquid-nitrogen-cooled CCD camera (Teledyne Princeton Instruments, PyLoN). Each HD-SFG measurement was performed under a N_2_ atmosphere by using the ssp polarization combination, where ssp denotes s-polarized SFG light, s-polarized visible light, and p-polarized mid-IR light.

Approximately 18 μL of the 10 mg/mL polymer solution was spread onto the water surface in a circular trough (diameter ≈ 5 cm). The mixture contains 1% v/v (∼0.98 wt %) of oleyl alcohol relative to toluene. To minimize thermal effects and avoid distortion of the polymer monolayer, the trough was gently rotated during measurements. Using a Langmuir–Blodgett trough (Biolin Scientific, KSV NIMA LB), the surface pressure of the monolayer under the specified conditions was determined to be 36.3 mN m^–1^ for the PB-*b*-PEO monolayer and 30.5 mN m^–1^ for the polymer monolayer with oleyl alcohol added.

### Fluorescence Correlation Spectroscopy (FCS)

Fluorescence Correlation Spectroscopy experiments were performed on a commercial device, an LSM 880 (Carl Zeiss, Jena, Germany). The excitation was done with the 543 nm line of a HeNe laser focused into the studied samples through a C-Apochromat 40×/1.2 W water immersion objective (Carl Zeiss, Jena, Germany). The emission light was collected with the same objective and, after passing through a confocal pinhole, directed to a spectral detection unit (Quasar, Carl Zeiss) in which a detection range of 560–700 nm was selected. Eight-well polystyrene chambered cover glasses (Nunc Lab-Tek, Thermo Fisher Scientific, Waltham, MA, USA) were used as sample cells for the studied vesicle dispersions.

The measurements were performed on both oleyl alcohol polymersomes and toluene polymersomes, which were formed 3 days prior. The observation well was prepared with 250 μL of 300 mM NaCl solution; 5 μL of each polymersomes sample was added, followed by 0.5 μL of Nile red solution (1 μg/mL in DMSO). After sedimentation of the vesicles at the bottom of the chamber, they were first visualized with the confocal laser scanning mode of the microscope. Next, the confocal observation volume was positioned precisely over the polymersome membrane at the top (“North pole”) of a vesicle, and a series of 6 FCS measurements with a total duration of 60 s were performed. These experiments were repeated multiple times with different vesicles. The time-dependent fluctuations of the fluorescent intensity δ*I*(τ) were recorded and analyzed by an autocorrelation function *G*(τ) = 1 + δ*I*(τ)·δ*I*(τ + τ)>/<*I*(τ)*>*.^2^ The experimental autocorrelation curves obtained in this way were fitted with a theoretical model function for 2D membrane diffusion^38^

1 $$G\left(\tau \right)=1+\frac{1}{N}\left[1+\frac{f_{T}}{1-f_{T}}e^{-\tau /\tau_{T}}\right]\frac{1}{\left[1+\frac{\tau }{\tau_{D}}\right]}$$

Here, *N* is the average number of diffusing fluorescence species in the observation volume, *f*_T_ and τ_T_ are the fraction and the decay times of the triplet state, respectively, and τ_*D*_ is the lateral diffusion time of the dye, which is directly related to its diffusion coefficient, *D*, through: $\tau_{D}=\frac{r_{0}^{2}}{4D}$ , where *r*_0_ is the radial dimensions of the confocal volume. The fits yielded the corresponding diffusion times of the Nile red dyes in the studied membranes.

### Fluorescence Recovery after Photobleaching (FRAP) Experiment

The FRAP experiment was conducted using a confocal laser scanning microscope (Leica TCS SP5, Wetzlar, Germany). The excitation was done with the 561 nm line of a HeNe laser focused into the samples through a C-Apochromat 63×/1.2 W water immersion objective (Carl Zeiss, Jena, Germany) to visualize and photobleach the dye. The emission light was collected with the same objective and, after passing through a confocal pinhole, directed to a spectral detection unit (Quasar, Carl Zeiss) in which a detection range of 611–769 nm was selected. The FRAP settings from LAS-X software were used. Eight-well ibidi chambered polymer bioinert coverslips (ibidi GmbH, Graefelfing, Germany) were used as observation chambers for the studied vesicles.

The observation wells were prepared with 250 μL of a 300 mM NaCl solution. Into 20 μL of polymersome dispersion prepared 5 days prior (TP) or 1 day prior (OAP), 1 μL of Nile red (10 μg/mL in DMSO) was added. 5 μL of each polymersome dispersion was added into the observation chambers and allowed to sediment. During the measurement, the focus was zoomed on one of the polymersomes, focusing on the cross section in the middle of the vesicle, identified by the position with the largest diameter. A ROI was chosen, a small section of the membrane, to be photobleached. The photobleaching was done in 10 frames at 102 ms intervals, using 100% laser power. Then, the polymersomes were continuously observed for 25 s at a 635 ms interval to record the fluorescence recovery speed. The result of five FRAP measurements is used for averaging in both the OAP and TP.

### Permeability of Protons through the Polymersome Membrane

8-Hydroxypyrene-1,3,6-trisulfonic acid (HPTS) pH probes are encapsulated inside polymersomes for proton permeability measurement. 10 μg mL^–1^ HPTS was added to the inner fluid of oleyl alcohol polymersomes, and 5 μg mL^–1^ HPTS was added to the inner fluid of toluene polymersomes. Both inner fluids and the outer fluid were adjusted to neutral pH, around 7.1–7.5. The polymersomes were prepared using microfluidics. Two exchange solutions were prepared to observe the pH change. The low-pH solution was prepared by adjusting the pH of a 200 mM NaCl solution to 6.0, whereas for the high pH solution, a 200 mM NaCl solution was adjusted to pH 9. The 300 mM NaCl outer fluid solution was used as a neutral pH control. Eight-well ibidi chambered polymer coverslips were used as observation chambers for the studied vesicles; 250 μL of the exchange solution was added into the observation well. Then, 5 μL of each polymersome was added into the three exchange solutions. Using a confocal laser scanning microscope (Leica TCS SP5, Wetzlar, Germany), the pH probe was visualized with a 458 nm line of an argon laser, with the emission detection range set to 510–530 nm. The polymersomes were observed right after the addition to the exchange solution.

### Permeability of Different Ions through the Polymersome Membrane

Coacervate encapsulated polymersomes using oleyl alcohol and toluene as the solvents were fabricated using droplet microfluidics. The composition of IF is mentioned in the Supporting Information in detail (Table S3). The final concentration of the coacervate pair is 2 mg/mL for AMC and 2 mg/mL for DEAE-Dextran. 400 mM glucose was used as the OF, and 10 mg mL^–1^ PB-*b*-PEO in either oleyl alcohol or toluene was used as the MF. Both OAP and TP were formed some days before to ensure complete dewetting has occurred. Into the observation wells, 250 μL of the salt solution (300 mM MgCl_2_, 200 mM CaCl_2_, 500 mM NaCl, 500 mM KCl, 500 mM LiCl), followed by the addition of either 1 μL of OAP or TP. Osmotically balanced salt solutions were used (Table S4). Next, they were imaged at regular time intervals on a confocal microscope (Leica TCS SP5, Wetzlar, Germany) or a bright-field microscope.

### Polymersomes as Microreactors: Permeability of Glucose through the Polymersome Membrane

To perform a glucose permeability test, 24 mM of NAD^+^, 2 U mL^–1^ of GDH, and 10 wt % dextran dissolved in 100 mM potassium phosphate buffer (pH 6.9) were encapsulated inside oleyl alcohol and toluene polymersomes using microfluidics. The middle fluid contained polymers dissolved in the respective solvent described in the previous section. For the outer fluid, 250 mM NaCl was used. For the reaction, an exchange solution containing 100 mM glucose and 150 mM NaCl was prepared. Eight-well ibidi chambered polymer coverslips were used as observation chambers for the studied vesicles. The observation chamber was prepared with 250 μL of the exchange solution. For control, 250 μL of the outer fluid was used. 5 μL of polymers was added into each solution to start the reaction. The GDH enzyme reaction was observed using the DAPI filter, in which the excitation and emission wavelengths overlap with those of NADH. Therefore, the generation of NADH in the event of GDH reaction can be confirmed. The system was observed for 60 min with a 20 min time interval.

## Supplementary Material



## Notes

Additional data, time-lapse optical images of dewetting of double emulsions to form polymersomes, confocal images of polymersomes, additional FRAP, FCS, NMR, and HPLC data, kinetics data for transmembrane diffusion of protons, time-lapse optical images of coacervate dissolution inside polymersomes, critical ionic strength for coacervate dissolution in bulk, and tables for the inner fluid compositions and osmotic pressures of the salt solutions used in the coacervate dissolution experiments (DOCX)
