# Supplementary material for: Cosurfactant-Induced Disorder in Polymersome Membrane Enhances Diffusion of Cargo Molecules
Source: ACS Nano. 2026 Apr 29;20(18):13706–17. doi: 10.1021/acsnano.6c00963 (PMC13173656; doi:10.1021/acsnano.6c00963)
Supplement: Supplementary file 1 [file nn6c00963_si_001.docx]

**Cosurfactant-Induced Disorder in Polymersome Membrane Enhances Diffusion of Cargo Molecules**

Gabrielle A. Ong^†^, Priyanka Sharan^†^, Robert Graf, Kaloian Koynov, Yucong Chen, Arsh S. Hazrah, Katharina Landfester*

Max Planck Institute for Polymer Research, Ackermannweg 10, 55128 Mainz, Germany

E-mail: [landfest@mpip-mainz.mpg.de](mailto:landfest@mpip-mainz.mpg.de)


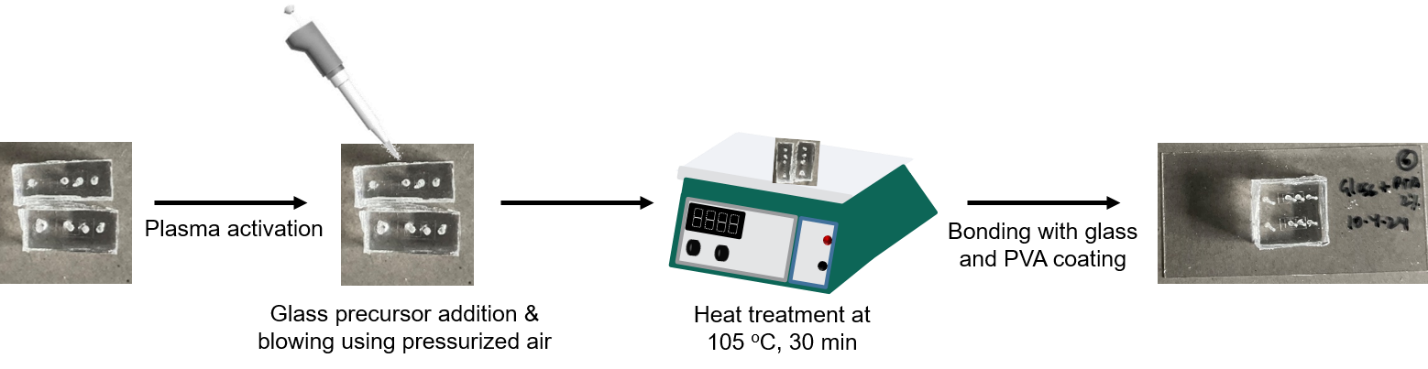


Figure S1: Schematic illustration of glass coating of the PDMS channels and chip production.

a.

Figure S2: a. Schematic illustration of microfluidic production of double emulsions. After solvent removal the double emulsions transform to polymersomes. Time-lapse optical images for the solvent removal in case of b. Oleyl alcohol and c. Toluene polymersomes. In case of oleyl alcohol, the dewetting occurs within 1 minute of production.

c.

b.


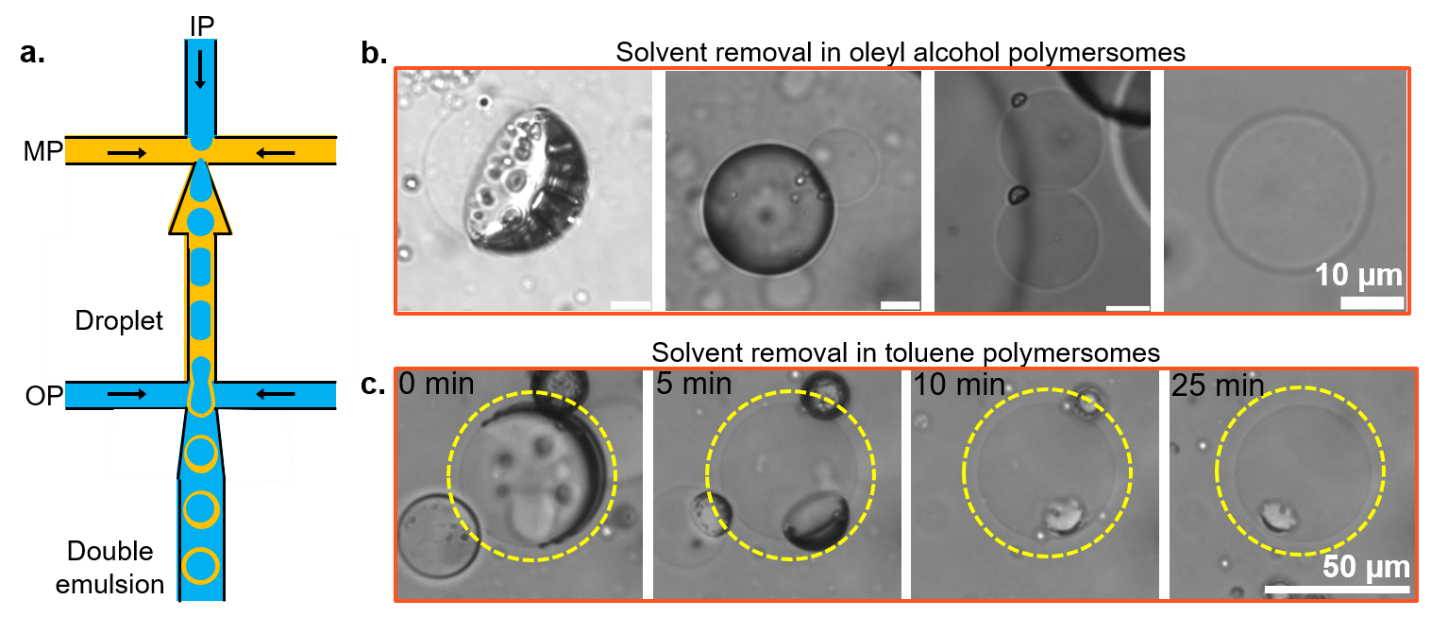


c.

b.

a.


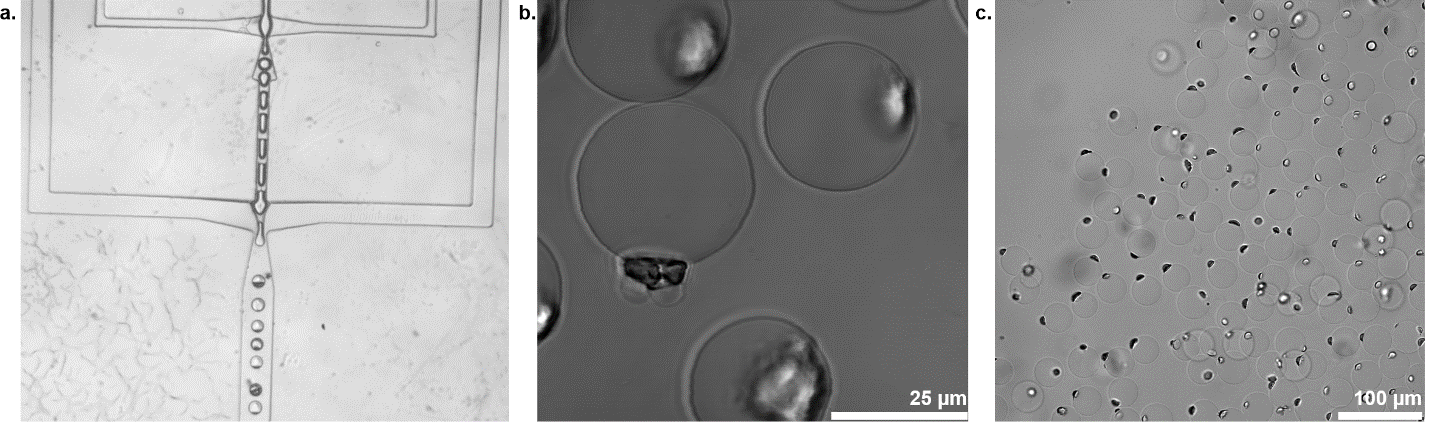


Figure S3: a. Production of double emulsions with toluene as the solvent. Image was acquired using a high-speed camera b, c. Toluene polymersomes.


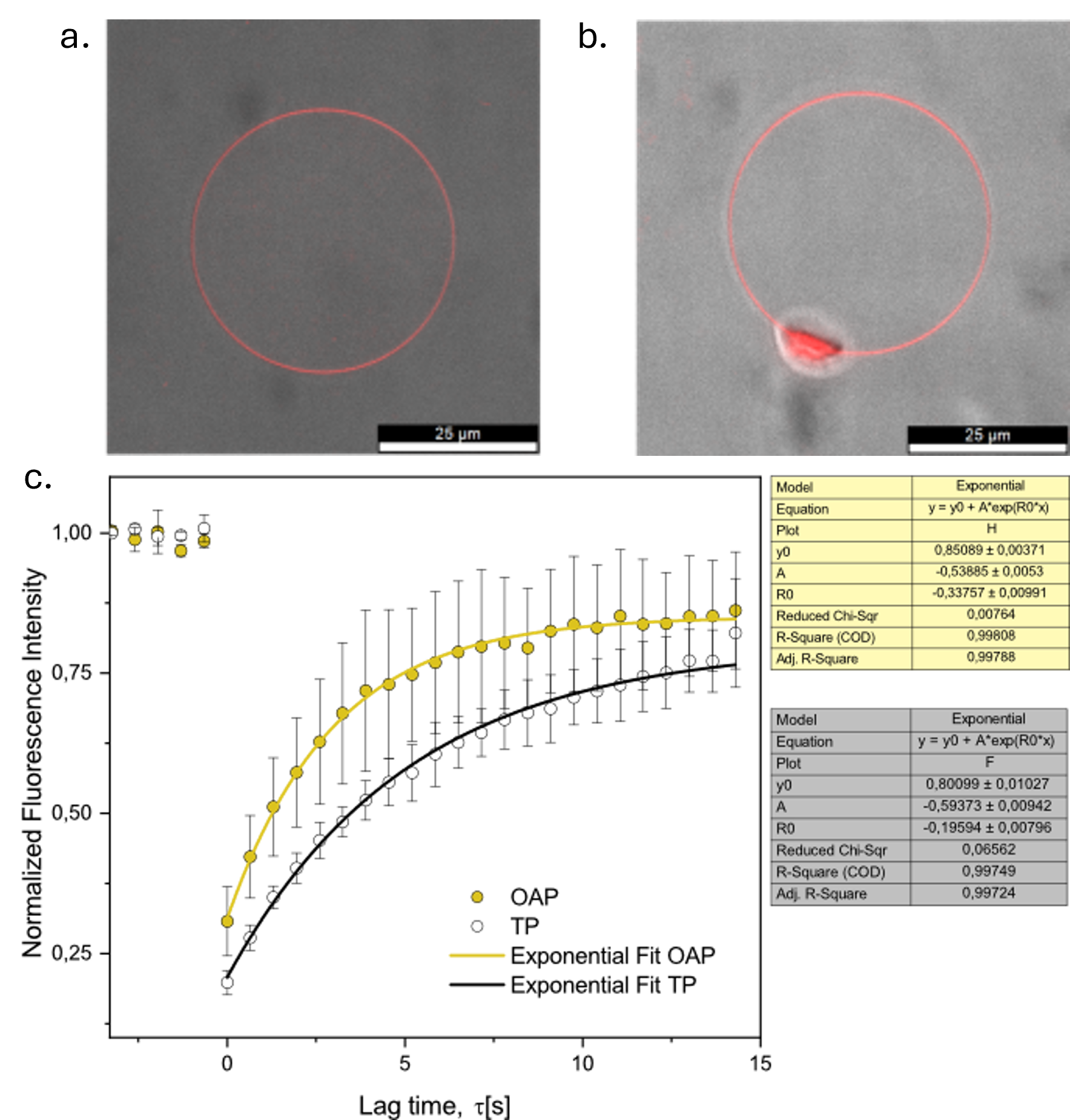


Figure S4: Confocal microscope image of (a) oleyl alcohol polymersomes and (b) toluene polymersomes. (c) FRAP measurement with error bars. Fluorescence intensity averaged for 6 polymersomes from 2 independent batch.


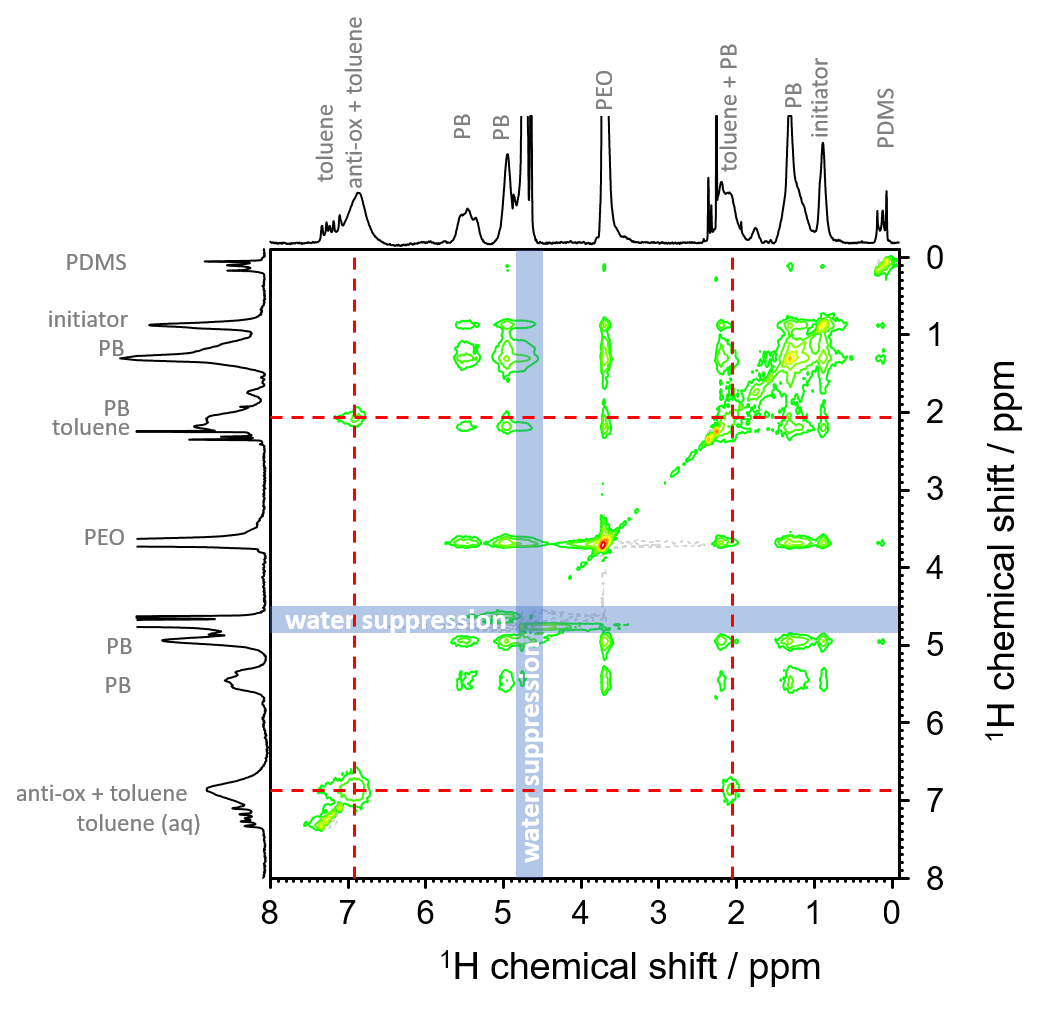


Figure S5: 2D NOESY NMR spectrum of a polymersome sample from toluene recorded with 300 ms NOE mixing delay. The aromatic protons of toluene in the polymer phase show correlation signals only with their neighboring methyl protons, but no significant correlations with the polymer signals as indicated by the dashed red lines. In contrast, the PDMS contaminations, extracted from the microfluidic device by toluene, are in close spatial proximity to all polymer sites, indicating that these PDMS particles are located in the polymersome membrane and not in the aqueous phase.

b.

a.


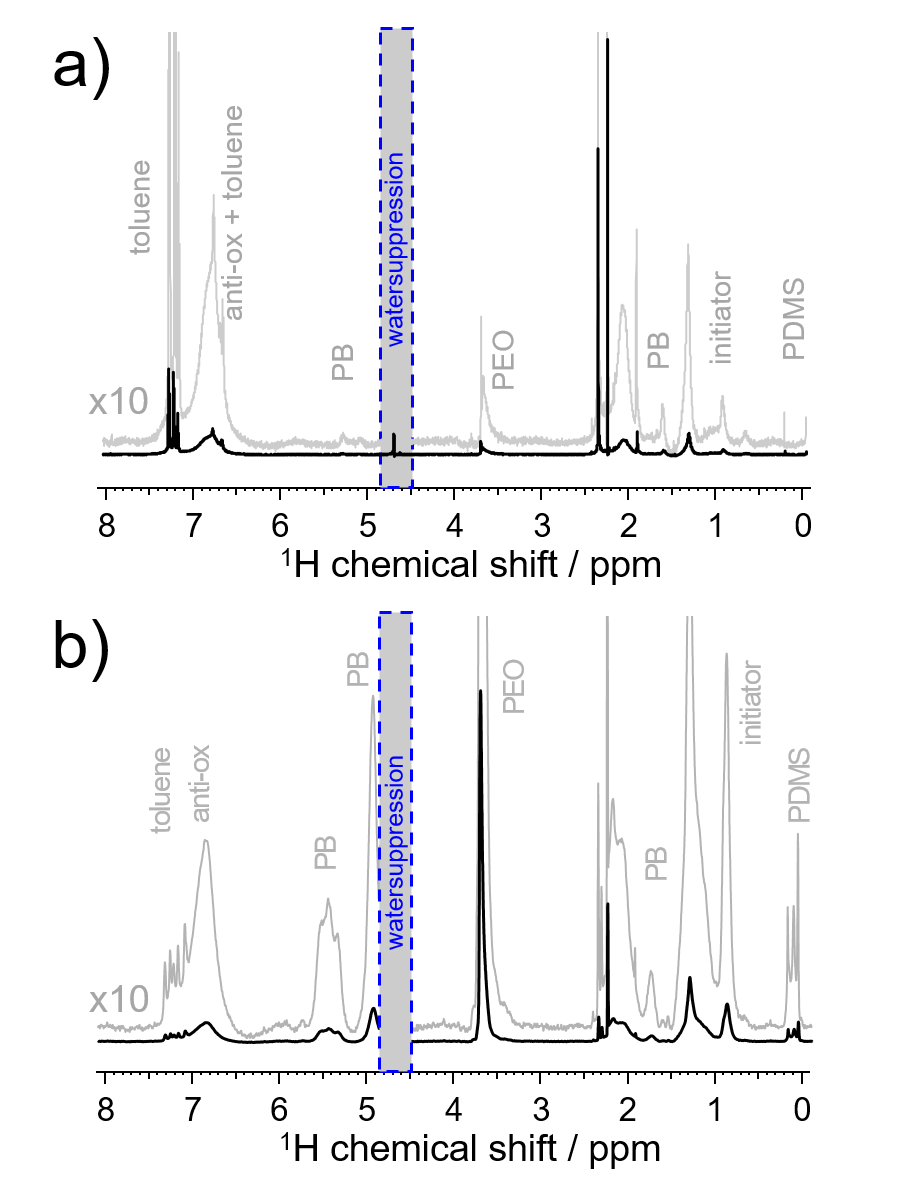


Figure S6: HR-MAS NMR spectra of toluene polymersomes taken (a) immediately after microfluidic formation, where the toluene peak can be found at around 7.15-7.3 ppm and (b) after two weeks of incubation in room temperature open to air. The toluene peak has shifted to lower ppm (7.09-7.3 ppm) indicating the presence of toluene in D_2_O. Anti-oxidant peak at around 6.8 ppm is found in the polymer, as we use commercial polymer and requires stabilizing agent due to the double bond of the butadiene group. Initiator used in the polymer synthesis resulted in a peak at around 0.8 ppm.


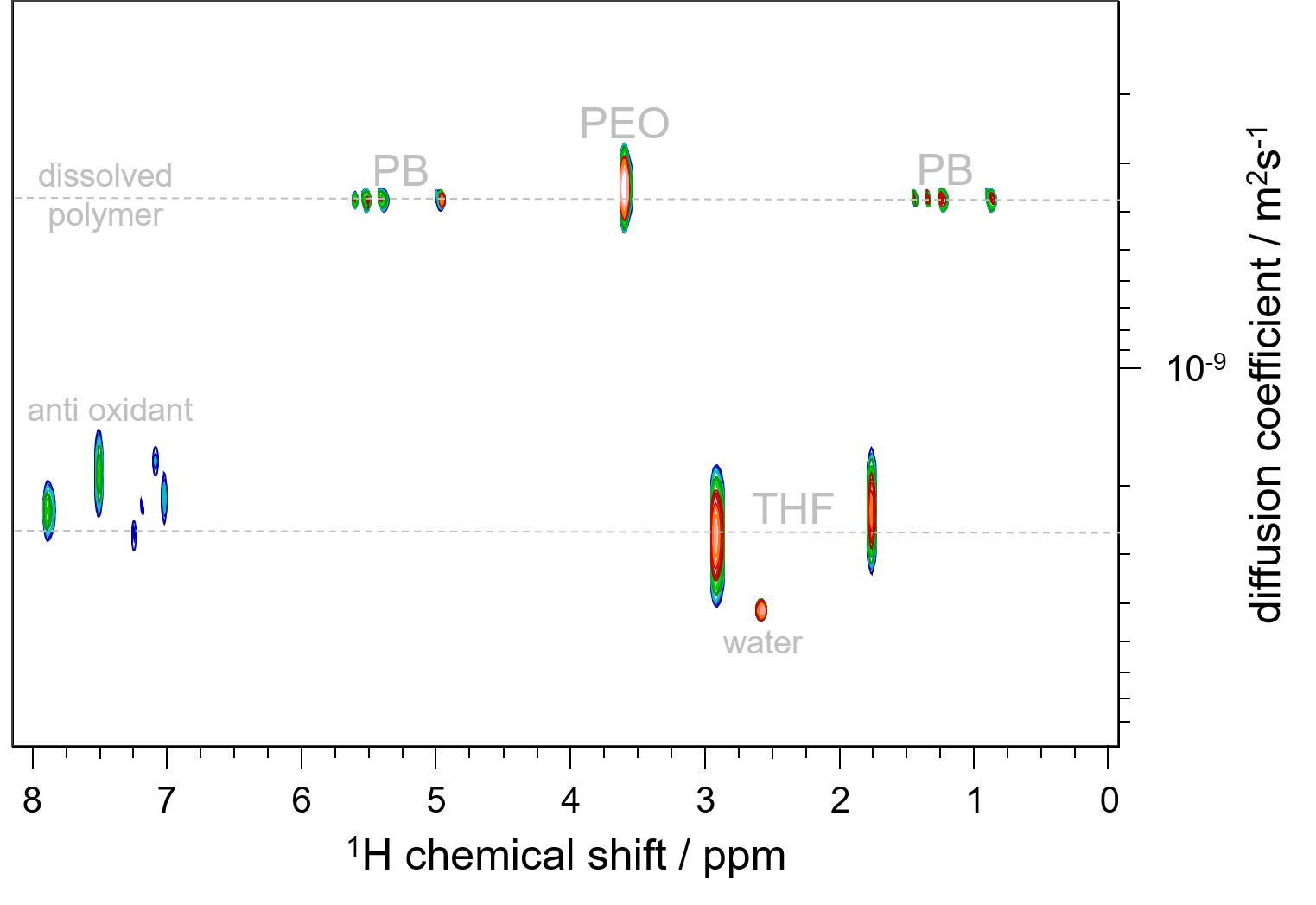

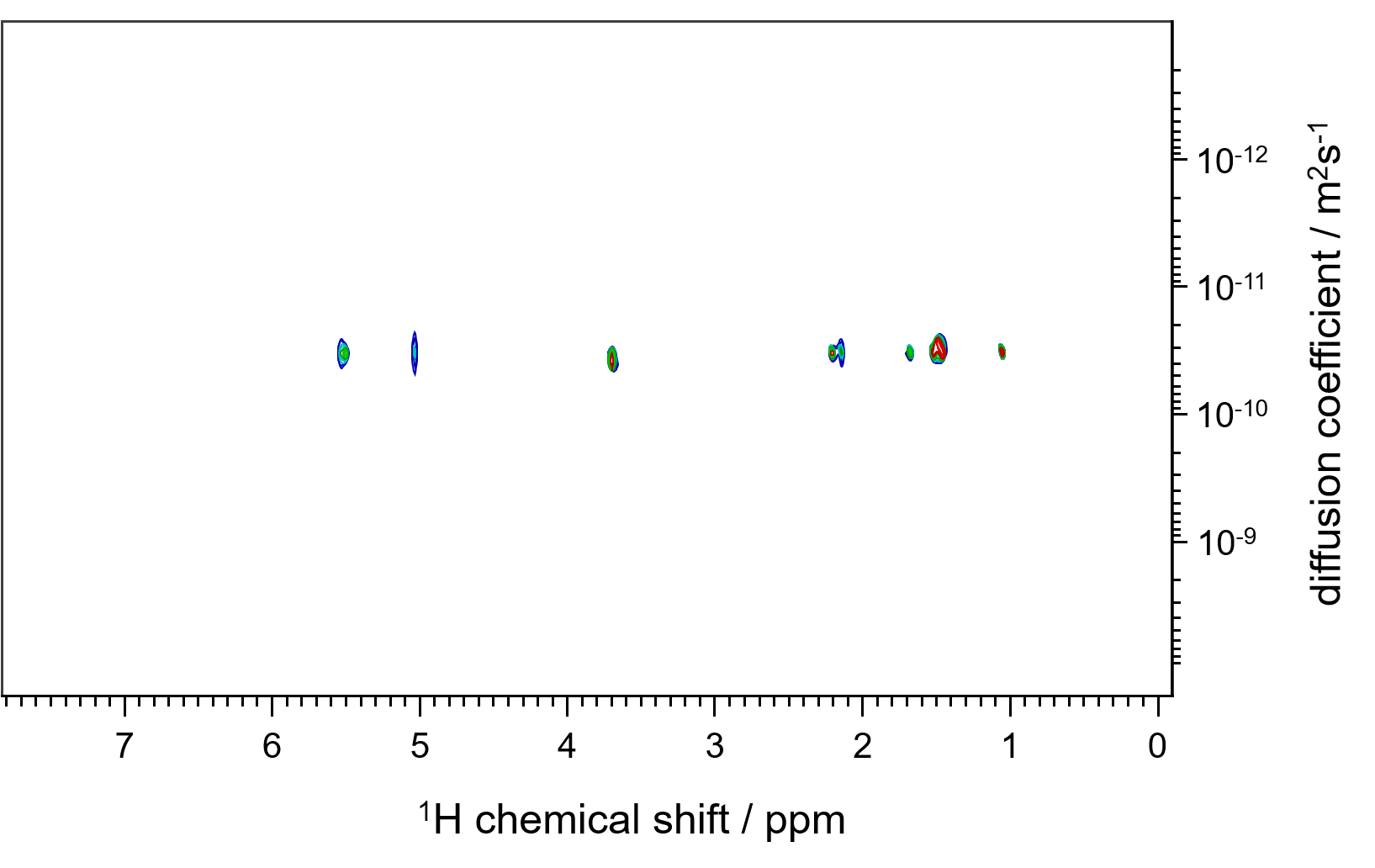


b.

a.

Figure S7: DOSY spectra of (a) oleyl alcohol in bulk and (b) polymer in THF.


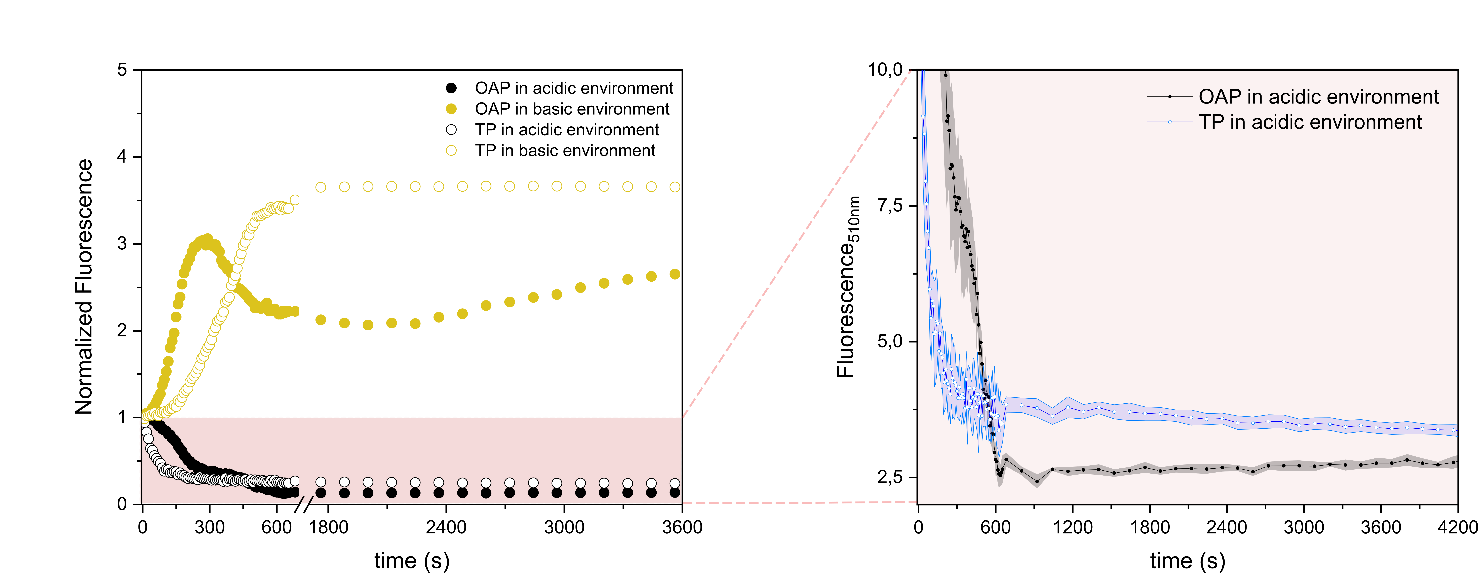


Figure S8: Fluorescence of pH probe in polymersomes. The polymersomes are incubated for one hour in acidic or basic environment. In basic environment, the OAP reach its highest fluorescence intensity peak within five minutes of incubation, indicating pH increase within the time frame. Whereas for TP, the fluorescence continues to increase until a plateau after 30 minute (1800 s). Looking at a zoomed-in plot on the right, the OAP fluorescence decrease significantly within 10 minutes of incubation in acidic environment. This indicates pH decrease inside the polymersomes, which then plateau. For TP, the fluorescence decrease drastically within 10 minutes, but then continues to decrease even after more than one hour of incubation. The result suggests slow and continuous proton diffusion through TP membrane. (N=10)

d.

e.

f.

c.

b.

a.


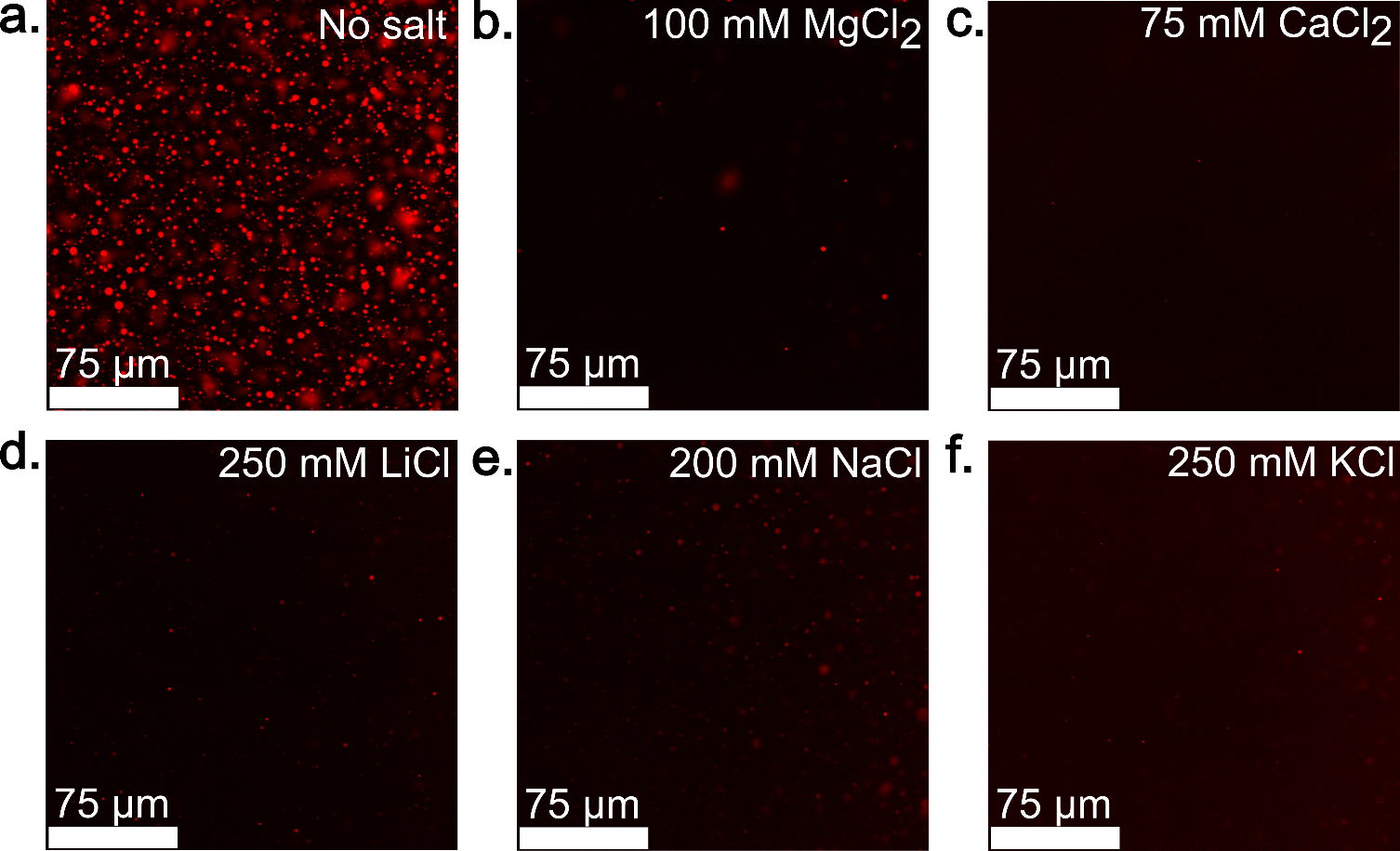


Figure S9: Complex coacervate droplets of carboxy-amylose (amylose-COOH) and diethylaminoethyl-dextran (DEAE-dextran) in different conditions on glass slide: a. No salt, b. 100 mM MgCl_2_, bc. 75 mM CaCl_2_, d. 250 mM LiCl, e. 200 mM LiCl, f. 250 mM KCl.

Figure S10a: Coacervate encapsulated oleyl alcohol Polymersomes in 300 mM MgCl_2_ solution.


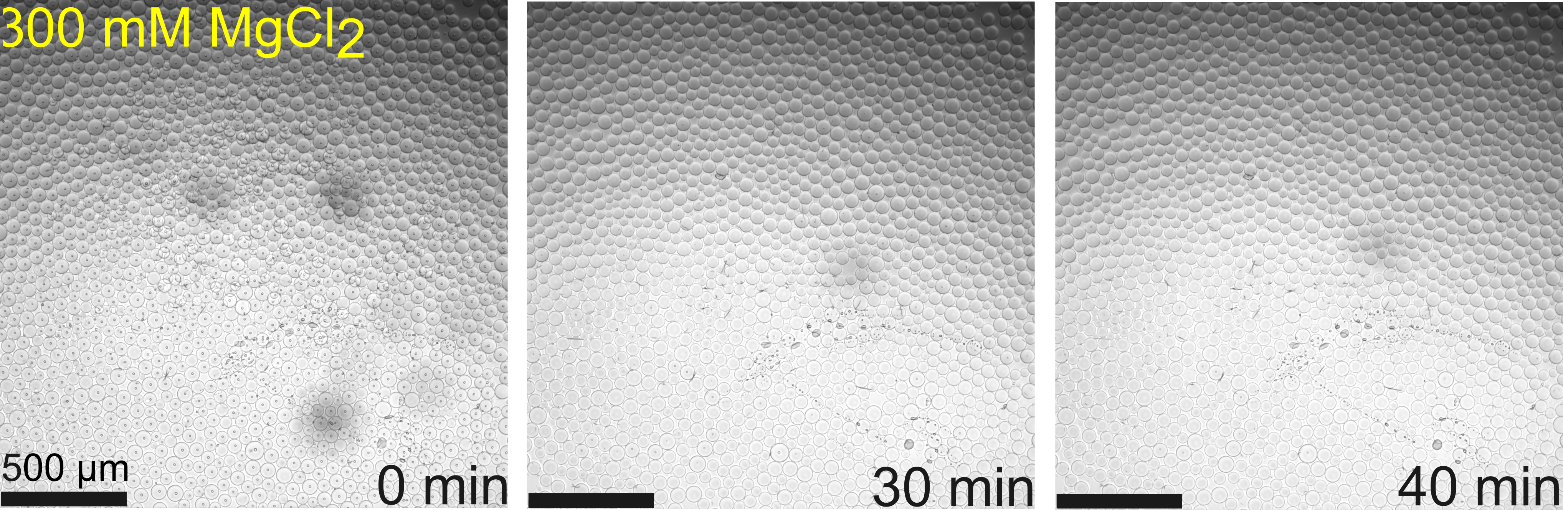


Figure S10b: Coacervate encapsulated oleyl alcohol Polymersomes in 200 mM CaCl_2_ solution.


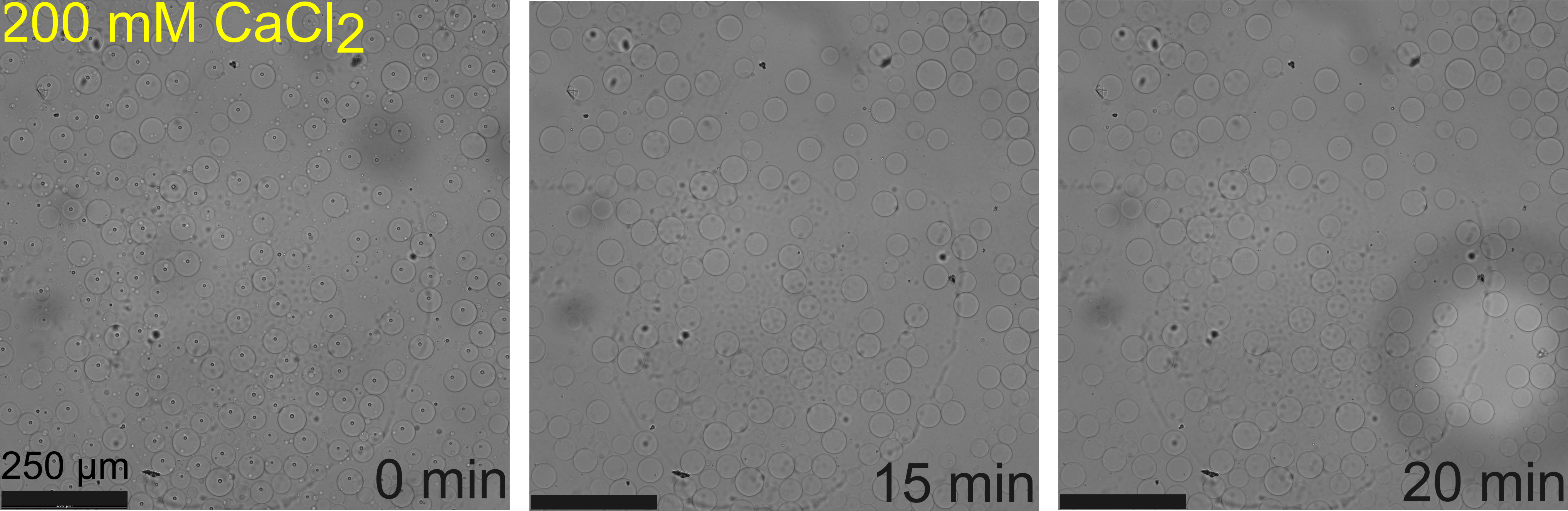


Figure S10c: Coacervate encapsulated oleyl alcohol Polymersomes in 500 mM LiCl solution.


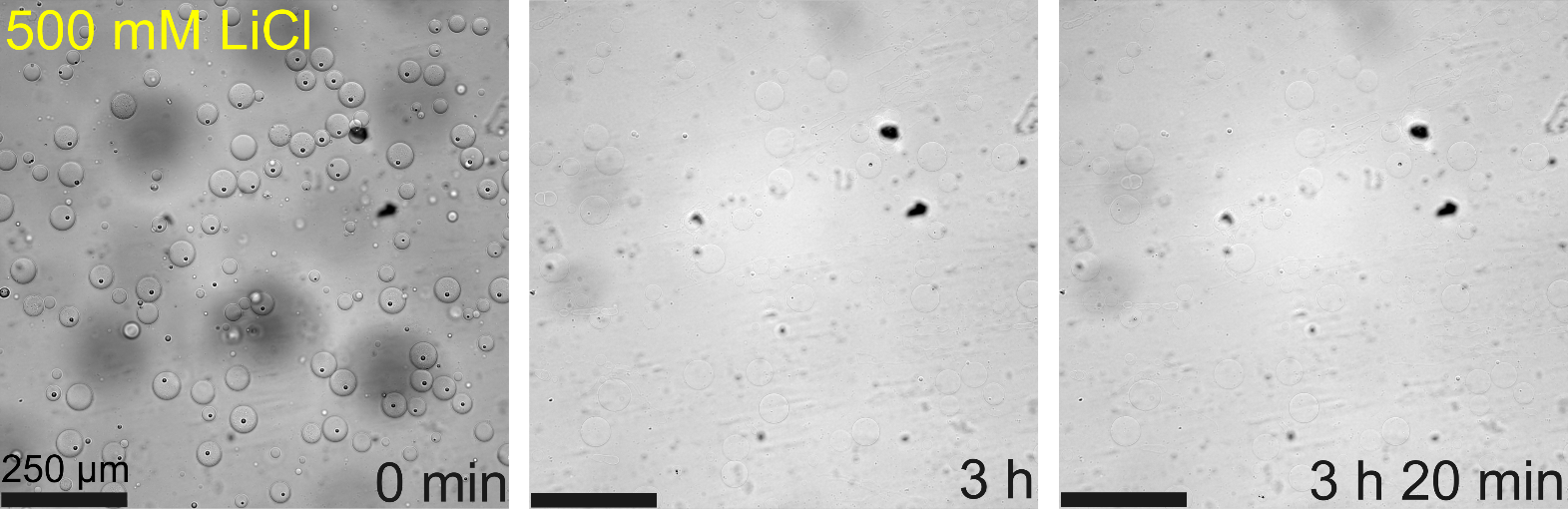


Figure S10d: Coacervate encapsulated oleyl alcohol Polymersomes in 300 mM NaCl solution.


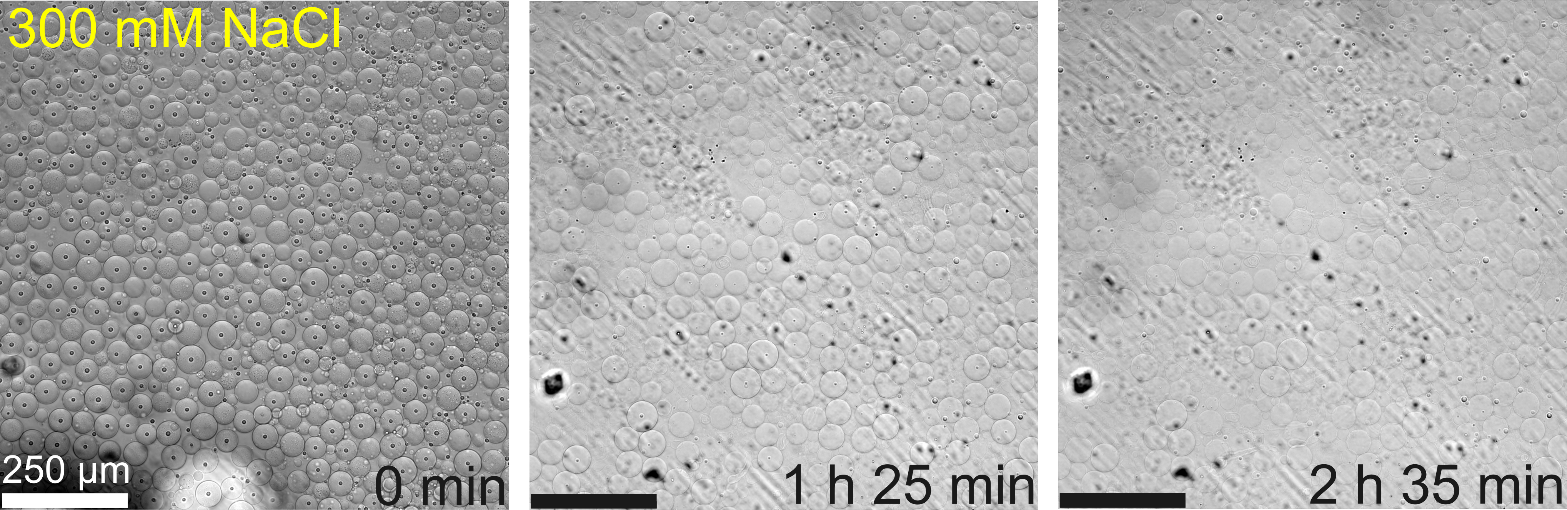


Figure S10e: Coacervate encapsulated oleyl alcohol Polymersomes in 300 mM KCl solution.


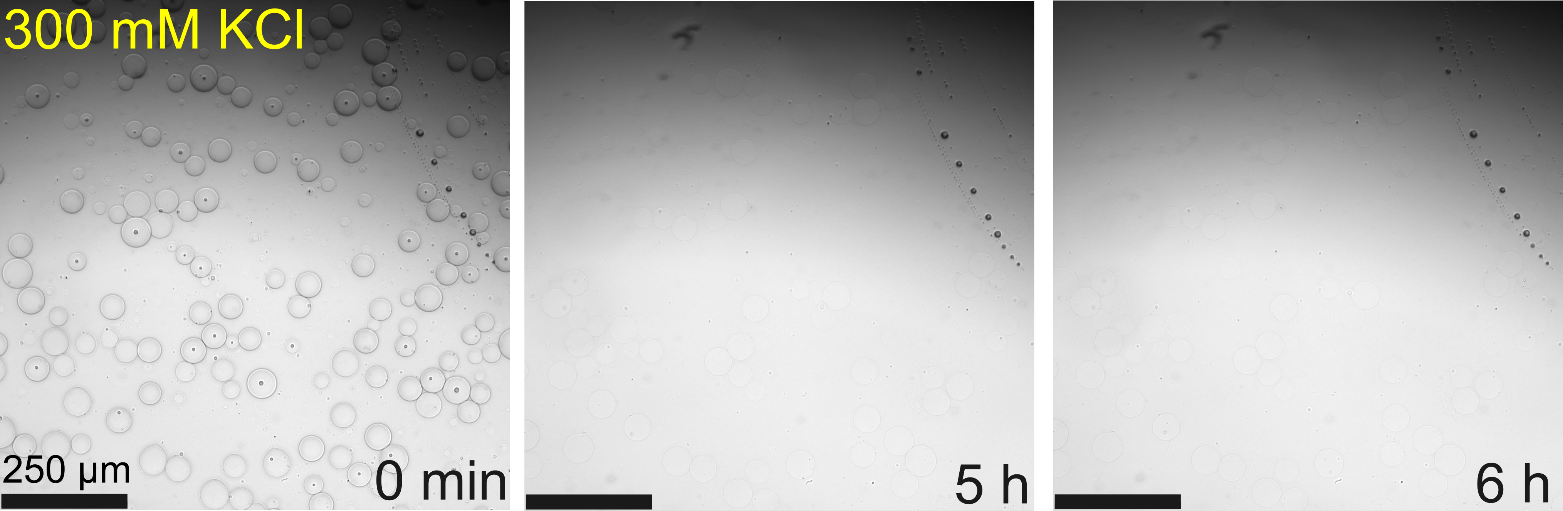


Figure S11a: Coacervate encapsulated toluene Polymersomes in 300 mM MgCl_2_ solution. Scale bar: 250 µm.


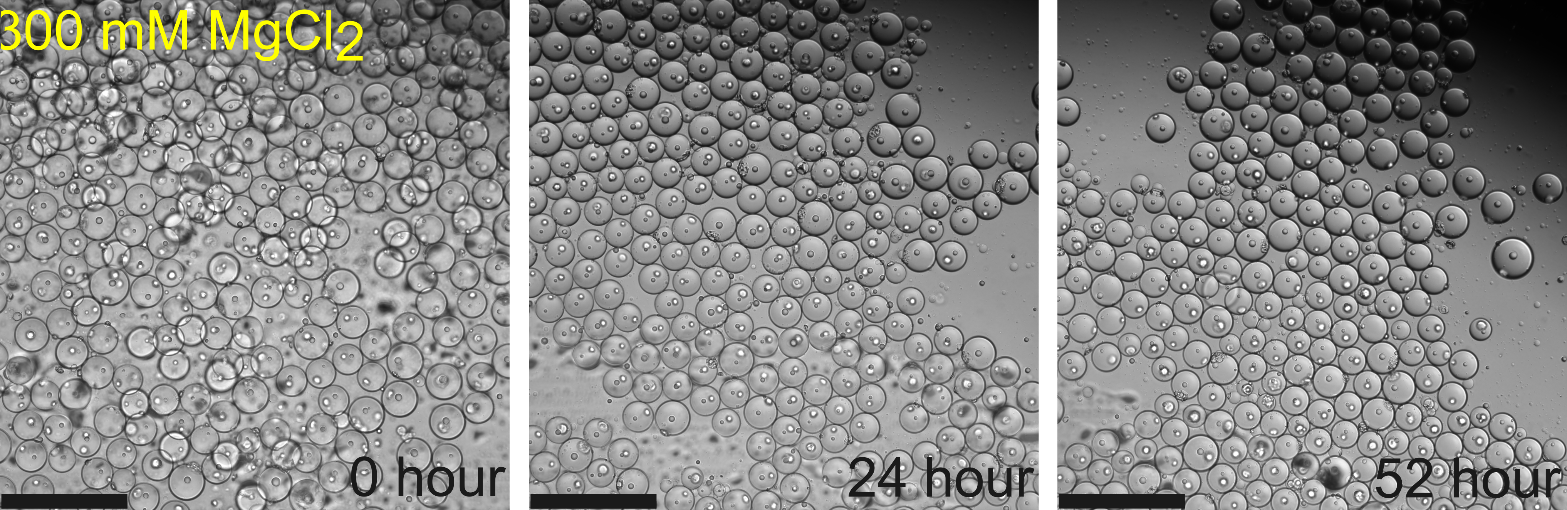


Figure S11b: Coacervate encapsulated toluene Polymersomes in 200 mM CaCl_2_ solution. Scale bar: 250 µm.


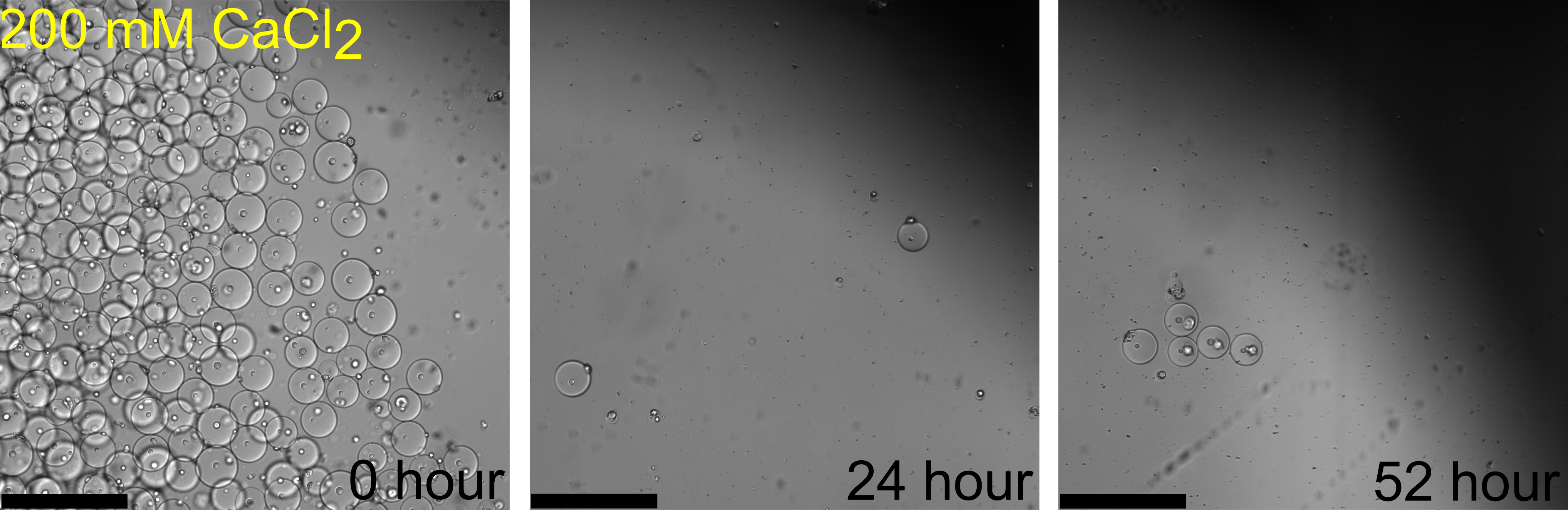


Figure S11c: Coacervate encapsulated toluene Polymersomes in 500 mM LiCl solution. Scale bar: 250 µm.


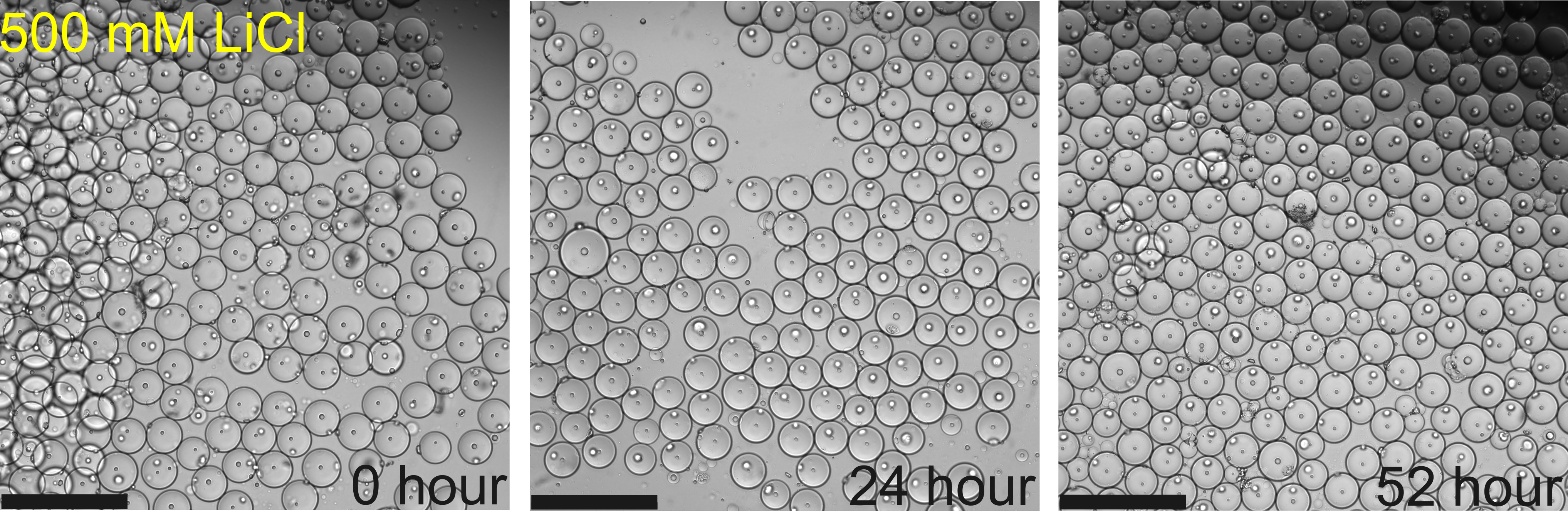


Figure S11d: Coacervate encapsulated toluene Polymersomes in 300 mM NaCl solution. Scale bar: 250 µm.


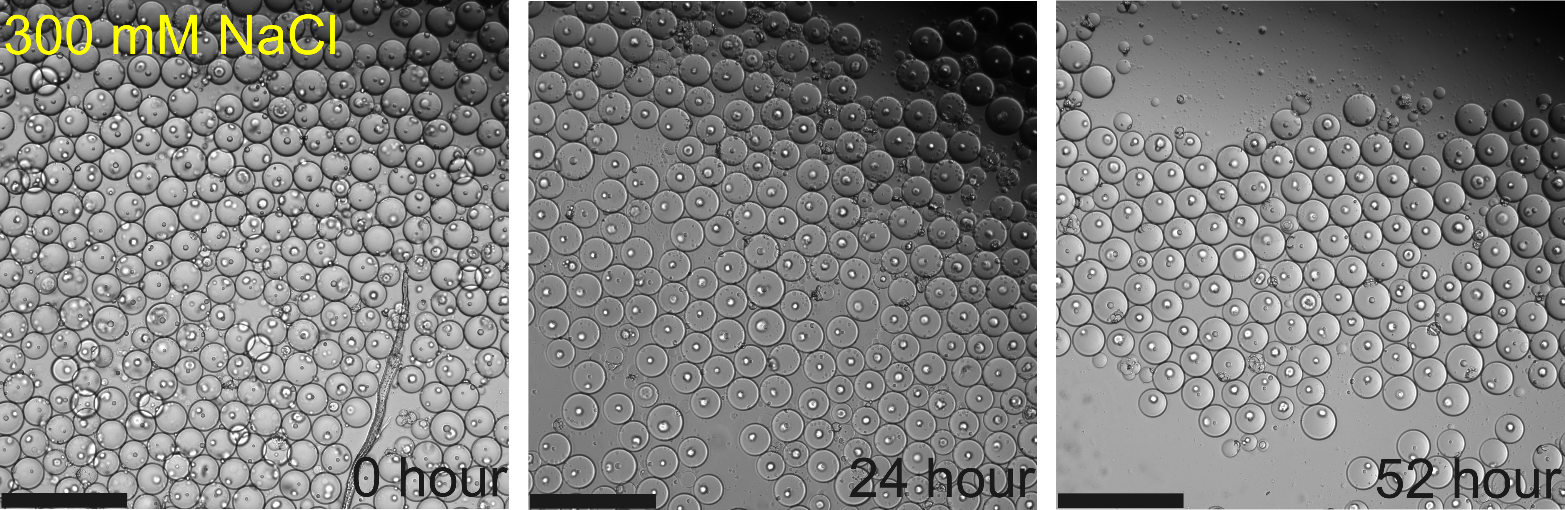


Figure S11e: Coacervate encapsulated toluene Polymersomes in 300 mM KCl solution. Scale bar: 250 µm.


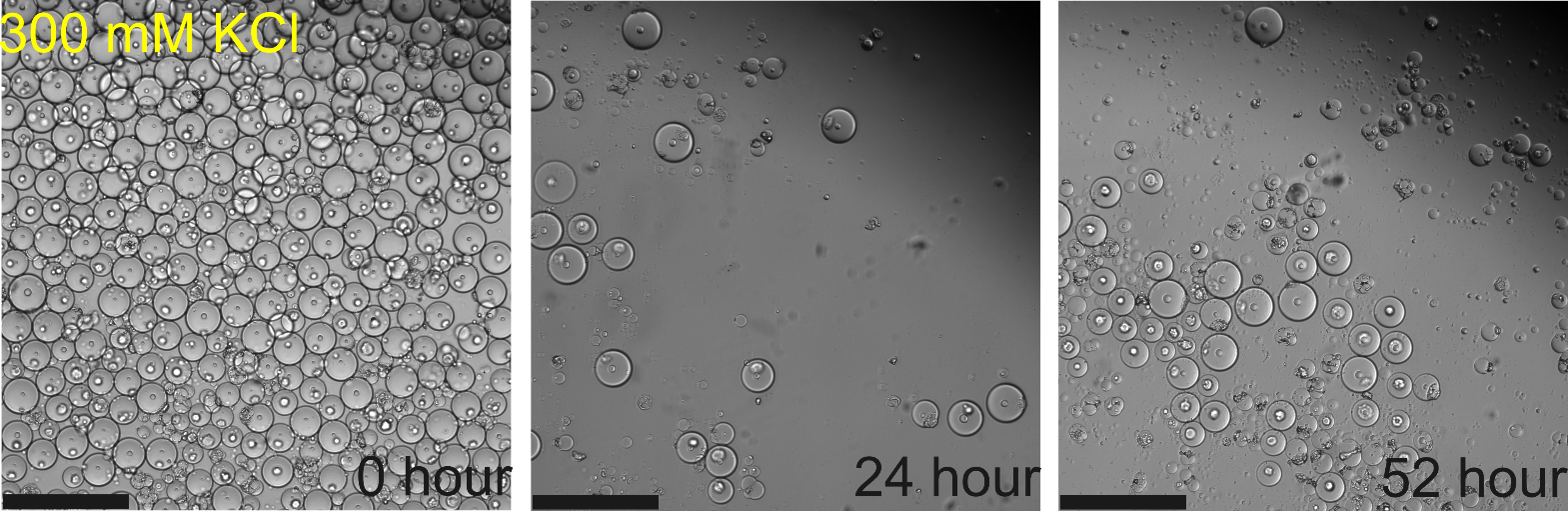


Figure S12: Critical ionic strength for coacervate dissolution.


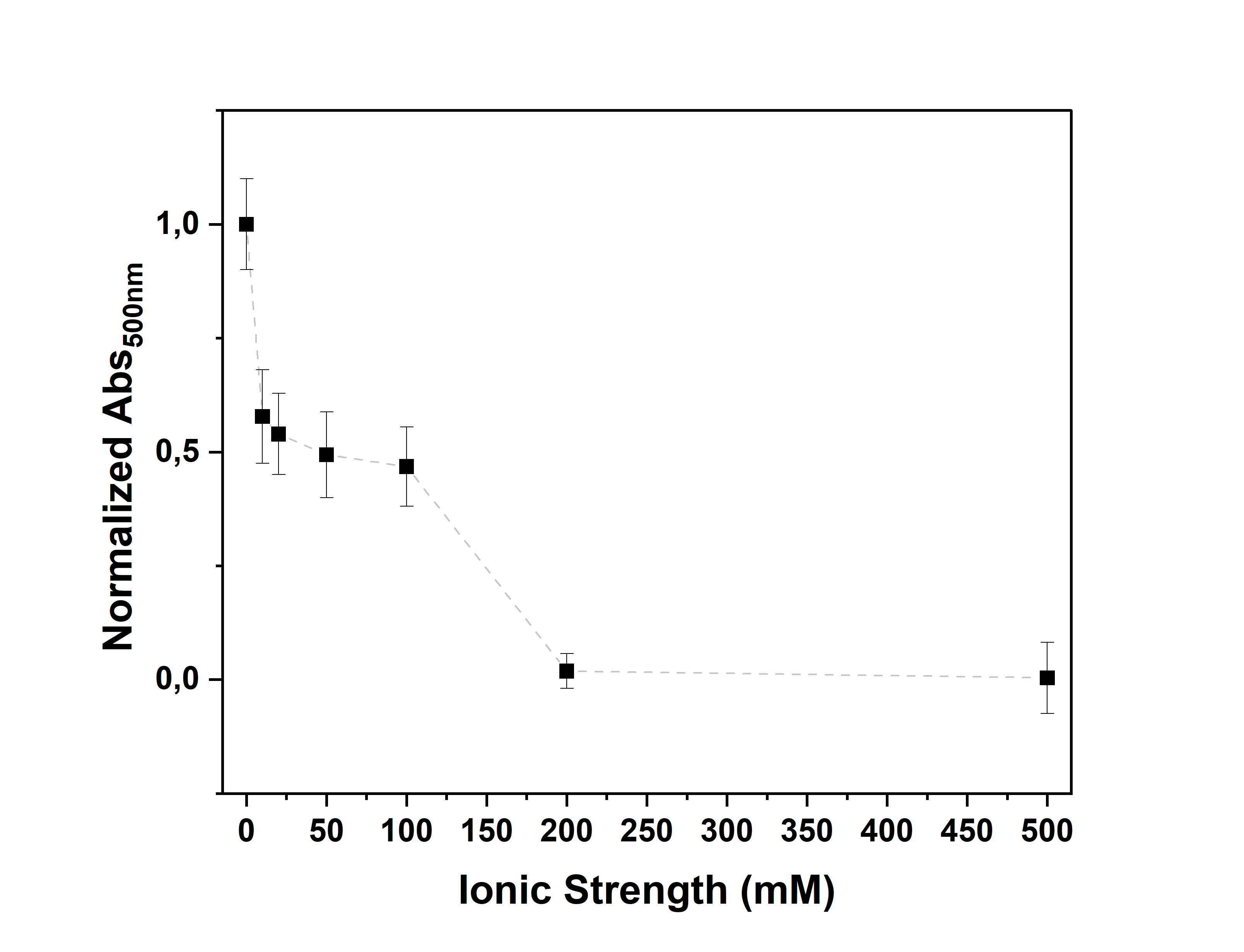


| Sample | Sample Conc. g/l in THF | Inj. Volume µL | UV Surface 200 nm | ELSD Surface | UV Conc. Solvent g/l | UV Solid % |
| --- | --- | --- | --- | --- | --- | --- |
| OAP1 | 1.99 | 5 | 528.315 | 9.87 e^-2^ | 0.14773714 | 6.258 |
| OAP2 | - | 5 | 523.138 | 8.68 e^-2^ | - | - |
| TP1 | 1.58 | 10 | 4.95847 | - | 0.00070 | 0.044 |
| TP2 | - | 10 | 4.96496 | - | - | - |

Table S1: HPLC result of the two different polymersomes system.

|  | OAP | TP |
| --- | --- | --- |
| Vesicle 1, Experiment 1 | 1520 ± 150 µs | 4200 ± 150 µs |
| Vesicle 1, Experiment 2 | 1780 ± 120 µs | 3400 ± 120 µs |
| Vesicle 1, Experiment 3 | 1620 ± 110 µs | 4510 ± 140 µs |
| Vesicle 2, Experiment 1 | 1560 ± 90 µs | 4020 ± 200 µs |
| Vesicle 2, Experiment 2 | 1790 ± 150 µs | 4220 ± 170 µs |

Table S2:  The diffusion time of Nile Red in OAP and TP. The error bars are the statistical errors from these 6 time 10s measurements.

| Sample | Concentration | V (uL) |
| --- | --- | --- |
| 1 M Sucrose | 400 mM | 20 |
| 20 mg/mL AMC in 5 mM HEPES | 2 mg/mL | 5 |
| 20 mg/mL DEAE-Dextran in 5 mM HEPES | 2 mg/mL | 5 |
| 1 M HEPES | 5 mM | 0.25 |
| 2 mg/mL DEAE-Dextran-RITC | 0.05 mg/mL | 1 |
| MQ water | - | 18.75 |

Table S3: Composition of inner fluid used in the coacervates experiments

| Sample | Osmotic pressure (mOsmol/Kg) | Ionic Strength  (mM) |
| --- | --- | --- |
| 400 mM Glucose (Outer fluid) | 397 | - |
| 400 mM Sucrose + coacervates (Inner fluid) | 411 | - |
| 300 mM MgCl_2_ | 873 | 750 |
| 200 mM CaCl_2_ | 477 | 500 |
| 500 mM LiCl | 512 | 500 |
| 300 mM NaCl | 592 | 300 |
| 300 mM KCl | 526 | 300 |

Table S4: Osmotic pressures and ionic strength of the inner, outer fluid and salt solutions used in coacervates experiments
